# Correction: Mind the gap: What explains the poor-non-poor inequalities in severe wasting among under-five children in low- and middle-income countries? Compositional and structural characteristics

**DOI:** 10.1371/journal.pone.0253831

**Published:** 2021-06-23

**Authors:** Adeniyi Francis Fagbamigbe, Ngianga-Bakwin Kandala, Olalekan A. Uthman

There are errors in the Funding statement. The correct Funding statement is as follows: AFF was supported by the Consortium for Advanced Research Training in Africa (CARTA). CARTA is jointly led by the African Population and Health Research Center and the University of the Witwatersrand and funded by the Carnegie Corporation of New York (Grant No—B 8606.R02), Sida (Grant No: 54100029), and the DELTAS Africa Initiative (Grant No: 107768/Z/15/Z).
