# Supplementary material for: Mind the gap: What explains the poor-non-poor inequalities in severe wasting among under-five children in low- and middle-income countries? Compositional and structural characteristics
Source: PLoS One. 2020 Nov 3;15(11):e0241416. doi: 10.1371/journal.pone.0241416 (PMC7608875; doi:10.1371/journal.pone.0241416)
Supplement: S1 Table — (DOCX) [file pone.0241416.s001.docx]

**Mind the Gap: What explains the poor-non-poor inequalities in severe wasting among under-five children in Low- and Middle-Income countries? Compositional and structural characteristics**

*Fagbamigbe, A. F.^1,2^, Kandala, N. B.^3^ & Uthman A. O.^2^

S1 : Detailed decomposition analysis

Test of homogeneity of Odds Ratios of severe wasting in countries with pro-poor inequalities

-------------------------------------------------------------------------------

country | Odds Ratio chi2(1) P>chi2 [95% Conf. Interval]

----------+--------------------------------------------------------------------

Angola | 1.965010 7.69 0.0055 1.20803 3.19633

Banglade | 1.560333 10.35 0.0013 1.18725 2.05065

Burundi | 2.932112 14.97 0.0001 1.65557 5.19295

CDR | 1.362143 5.17 0.0230 1.04239 1.77999

Cameroun | 3.314145 27.16 0.0000 2.05474 5.34546

Ethiopia | 1.841293 24.45 0.0000 1.44010 2.35426

Ghana | 2.593361 4.37 0.0366 1.02532 6.55942

India | 1.321868 299.95 0.0000 1.28065 1.36442

Kenya | 2.071787 25.68 0.0000 1.55339 2.76318

Lesotho | 6.342314 7.62 0.0058 1.40690 28.59122

Mali | 1.557305 10.13 0.0015 1.18294 2.05014

Mozambiq | 1.981869 18.58 0.0000 1.44331 2.72138

Namibia | 2.116439 4.77 0.0290 1.06283 4.21453

Niger | 1.276457 4.32 0.0378 1.01332 1.60792

Nigeria | 1.539260 79.20 0.0000 1.39875 1.69389

Pakistan | 2.333085 21.43 0.0000 1.61228 3.37615

Senegal | 1.828174 13.72 0.0002 1.32203 2.52810

Timor | 1.366375 12.75 0.0004 1.15039 1.62291

Uganda | 2.475533 11.42 0.0007 1.43691 4.26488

Zimbabwe | 1.749673 4.57 0.0325 1.04081 2.94132

-------------------------------------------------------------------------------

Mantel-Haenszel estimate controlling for country

----------------------------------------------------------------

Odds Ratio chi2(1) P>chi2 [95% Conf. Interval]

----------------------------------------------------------------

1.385718 528.45 0.0000 1.347541 1.424976

----------------------------------------------------------------

Test of homogeneity of ORs (approx): chi2(19) = 79.27

Pr>chi2 = 0.0000

The detailed decomposition analysis of factors associated with severe wasting in pro-poor countries

.

. *foreach num of numlist 5 10 14 16 21 22 24 31 34 {

. foreach num of numlist 1 / `=N' {

2. display "`num'"

3. oaxaca sam `ind' `com' if country == `num' , by(poor) logit pooled relax

4. local names : colfullnames e(b)

5. matrix k = e(b)

6. local lab: label country `num'

7. local country_first = "`lab'"

8. local country_reshape = "`country_reshape' `lab'"

9. local country_keep = "`country_keep' `lab'*"

10. *local lab = string(cot_)+`num'

. svmat k, name("`lab'")

11. }

1

(bint3 com_zsesc55 dropped from model 1)

(border1 dropped from model 2)

(bint3 dropped from pooled model)

(bint3 com_zsesc55 missing in model 1; assumed zero)

(border1 missing in model 2; assumed zero)

(bint3 missing in pooled model; assumed zero)

Blinder-Oaxaca decomposition Number of obs = 6001

Model = logit

Group 1: poor = 0 N of obs 1 = 2925

Group 2: poor = 1 N of obs 2 = 3076

------------------------------------------------------------------------------

| Robust

sam | Coef. Std. Err. z P>|z| [95% Conf. Interval]

-------------+----------------------------------------------------------------

overall |

group_1 | .0078632 .0016304 4.82 0.000 .0046677 .0110588

group_2 | .0162549 .0022778 7.14 0.000 .0117904 .0207193

difference | -.0083916 .0028012 -3.00 0.003 -.0138819 -.0029014

explained | -.0021469 .0031095 -0.69 0.490 -.0082414 .0039475

unexplained | -.0062447 .0045525 -1.37 0.170 -.0151674 .002678

-------------+----------------------------------------------------------------

explained |

mage2 | .0001803 .0001909 0.94 0.345 -.0001938 .0005545

mage3 | -.0002975 .000205 -1.45 0.147 -.0006992 .0001042

medu1 | -.0013011 .0011463 -1.14 0.256 -.0035478 .0009455

medu2 | -.0001313 .0001421 -0.92 0.356 -.0004098 .0001472

rural1 | .0041653 .0027227 1.53 0.126 -.0011711 .0095016

notwork | .0007759 .0005927 1.31 0.191 -.0003858 .0019376

media | -.0009592 .0013407 -0.72 0.474 -.003587 .0016685

water | -.0000838 .0006744 -0.12 0.901 -.0014056 .001238

toilety | -.0003509 .0009121 -0.38 0.700 -.0021386 .0014368

mar1 | -.0000406 .0000816 -0.50 0.618 -.0002005 .0001192

mar2 | -.0000246 .0001229 -0.20 0.841 -.0002654 .0002162

sex | .000027 .0000558 0.48 0.629 -.0000824 .0001364

age | -.0000434 .0000748 -0.58 0.562 -.00019 .0001033

weightm2 | 1.76e-06 .0000141 0.12 0.901 -.0000259 .0000295

weightm3 | -.0000202 .0000438 -0.46 0.644 -.0001061 .0000656

border1 | .0002044 .0001937 1.06 0.291 -.0001753 .0005841

border2 | .000089 .0001199 0.74 0.458 -.0001459 .0003239

border3 | -.0000316 .0000469 -0.67 0.500 -.0001235 .0000602

bint2 | -.0003939 .0003171 -1.24 0.214 -.0010154 .0002276

bint3 | 0 (omitted)

com_zsesc52 | .0003273 .000922 0.35 0.723 -.0014797 .0021343

com_zsesc53 | .0000827 .0002422 0.34 0.733 -.000392 .0005573

com_zsesc54 | -.00222 .0020697 -1.07 0.283 -.0062766 .0018366

com_zsesc55 | -.0021023 .0024139 -0.87 0.384 -.0068336 .0026289

-------------+----------------------------------------------------------------

unexplained |

mage2 | -.0041585 .0039687 -1.05 0.295 -.0119369 .0036199

mage3 | -.0020886 .0022988 -0.91 0.364 -.0065942 .0024169

medu1 | .0003864 .0025819 0.15 0.881 -.0046741 .0054469

medu2 | .0030734 .0027495 1.12 0.264 -.0023156 .0084623

rural1 | -.0046676 .0023707 -1.97 0.049 -.0093141 -.0000212

notwork | -.0009527 .0020846 -0.46 0.648 -.0050384 .0031331

media | -.0046599 .0042236 -1.10 0.270 -.012938 .0036182

water | .0002124 .002865 0.07 0.941 -.0054029 .0058277

toilety | -.0005787 .0012492 -0.46 0.643 -.0030272 .0018697

mar1 | .0000531 .0018056 0.03 0.977 -.0034858 .0035919

mar2 | .0070434 .0083516 0.84 0.399 -.0093255 .0234122

sex | -.0003767 .0026427 -0.14 0.887 -.0055563 .0048028

age | -.0014109 .0038429 -0.37 0.714 -.0089429 .0061211

weightm2 | .0003638 .0005195 0.70 0.484 -.0006545 .0013821

weightm3 | .0000886 .0004258 0.21 0.835 -.0007461 .0009232

border1 | .0009989 .001904 0.52 0.600 -.0027329 .0047306

border2 | -.0004413 .0017191 -0.26 0.797 -.0038108 .0029282

border3 | .0004775 .0015349 0.31 0.756 -.0025309 .0034859

bint2 | .0059904 .0046803 1.28 0.201 -.0031827 .0151635

bint3 | .0021986 .0021652 1.02 0.310 -.002045 .0064423

com_zsesc52 | -.0006656 .0015104 -0.44 0.659 -.0036258 .0022947

com_zsesc53 | .0001598 .0018129 0.09 0.930 -.0033935 .003713

com_zsesc54 | .0022981 .0024265 0.95 0.344 -.0024577 .007054

com_zsesc55 | .0013175 .0022964 0.57 0.566 -.0031834 .0058184

_cons | -.0109059 .0204435 -0.53 0.594 -.0509744 .0291626

------------------------------------------------------------------------------

2

(mar1 border1 dropped from model 1)

(mar1 bint2 dropped from model 2)

(mar1 border1 dropped from pooled model)

(mar1 border1 missing in model 1; assumed zero)

(mar1 bint2 missing in model 2; assumed zero)

(mar1 border1 missing in pooled model; assumed zero)

Blinder-Oaxaca decomposition Number of obs = 3777

Model = logit

Group 1: poor = 0 N of obs 1 = 2246

Group 2: poor = 1 N of obs 2 = 1531

------------------------------------------------------------------------------

| Robust

sam | Coef. Std. Err. z P>|z| [95% Conf. Interval]

-------------+----------------------------------------------------------------

overall |

group_1 | .0289403 .0035358 8.18 0.000 .0220102 .0358705

group_2 | .0496408 .0055402 8.96 0.000 .0387821 .0604994

difference | -.0207004 .0065724 -3.15 0.002 -.0335821 -.0078188

explained | .0020942 .0036485 0.57 0.566 -.0050567 .0092452

unexplained | -.0227946 .0070303 -3.24 0.001 -.0365738 -.0090155

-------------+----------------------------------------------------------------

explained |

mage2 | -.0000899 .0001277 -0.70 0.481 -.0003401 .0001603

mage3 | -.0000343 .0001678 -0.20 0.838 -.0003632 .0002946

medu1 | -.000716 .0011817 -0.61 0.545 -.003032 .0016001

medu2 | -.0020318 .0011004 -1.85 0.065 -.0041886 .000125

rural1 | -.0013191 .0019996 -0.66 0.509 -.0052383 .0026001

notwork | -.0005386 .0003489 -1.54 0.123 -.0012225 .0001452

media | .0020889 .0027501 0.76 0.448 -.0033013 .007479

water | 1.16e-06 .0003341 0.00 0.997 -.0006536 .000656

toilety | .0007165 .0016861 0.42 0.671 -.0025882 .0040213

mar1 | 0 (omitted)

mar2 | -.0000153 .0000699 -0.22 0.827 -.0001522 .0001217

sex | 2.12e-06 .0001717 0.01 0.990 -.0003344 .0003387

age | -.0000276 .0001294 -0.21 0.831 -.0002811 .0002259

weightm2 | -.0001144 .0001424 -0.80 0.422 -.0003935 .0001647

weightm3 | -.0004399 .0002304 -1.91 0.056 -.0008916 .0000118

border1 | 0 (omitted)

border2 | .0001647 .0002646 0.62 0.534 -.0003538 .0006833

border3 | -.000183 .0002932 -0.62 0.533 -.0007575 .0003916

bint2 | .0005043 .000575 0.88 0.380 -.0006227 .0016312

bint3 | -.0002196 .0004847 -0.45 0.650 -.0011697 .0007304

com_zsesc52 | -.0012567 .0016082 -0.78 0.435 -.0044086 .0018953

com_zsesc53 | -.000028 .0001258 -0.22 0.824 -.0002745 .0002186

com_zsesc54 | .0022414 .0017658 1.27 0.204 -.0012195 .0057024

com_zsesc55 | .0033892 .0035289 0.96 0.337 -.0035272 .0103057

-------------+----------------------------------------------------------------

unexplained |

mage2 | .0100204 .0075258 1.33 0.183 -.0047298 .0247706

mage3 | .0006772 .0019229 0.35 0.725 -.0030915 .004446

medu1 | .0030844 .0030142 1.02 0.306 -.0028234 .0089922

medu2 | .0022791 .0040036 0.57 0.569 -.0055678 .0101261

rural1 | .0196617 .0146873 1.34 0.181 -.009125 .0484483

notwork | -.0012433 .0103653 -0.12 0.905 -.0215589 .0190724

media | .0227166 .008797 2.58 0.010 .0054749 .0399584

water | -.0191472 .0438324 -0.44 0.662 -.1050571 .0667628

toilety | .0019212 .01064 0.18 0.857 -.0189328 .0227751

mar1 | 0 (omitted)

mar2 | -.0223906 .0552965 -0.40 0.686 -.1307697 .0859884

sex | -.0082932 .0068454 -1.21 0.226 -.02171 .0051235

age | -.0121741 .008477 -1.44 0.151 -.0287888 .0044405

weightm2 | .0015357 .0021878 0.70 0.483 -.0027523 .0058236

weightm3 | -.0013439 .0013307 -1.01 0.313 -.0039521 .0012643

border1 | -.0012874 .0056661 -0.23 0.820 -.0123928 .0098181

border2 | .0021612 .0075114 0.29 0.774 -.0125608 .0168833

border3 | .0040216 .0040661 0.99 0.323 -.0039478 .0119911

bint2 | -.0039842 .0036328 -1.10 0.273 -.0111042 .0031359

bint3 | -.0050086 .0114385 -0.44 0.661 -.0274276 .0174105

com_zsesc52 | -.0121107 .005699 -2.13 0.034 -.0232805 -.0009408

com_zsesc53 | -.0157225 .0094491 -1.66 0.096 -.0342423 .0027973

com_zsesc54 | -.0196852 .0119469 -1.65 0.099 -.0431008 .0037303

com_zsesc55 | -.0205183 .0148672 -1.38 0.168 -.0496574 .0086209

_cons | .0520352 .0870823 0.60 0.550 -.1186429 .2227134

------------------------------------------------------------------------------

3

(border1 dropped from model 1)

(notwork mar1 border1 dropped from model 2)

(model 2 has zero variance coefficients)

(bint3 dropped from pooled model)

(border1 missing in model 1; assumed zero)

(notwork mar1 border1 missing in model 2; assumed zero)

(bint3 missing in pooled model; assumed zero)

Blinder-Oaxaca decomposition Number of obs = 5853

Model = logit

Group 1: poor = 0 N of obs 1 = 3601

Group 2: poor = 1 N of obs 2 = 2252

------------------------------------------------------------------------------

| Robust

sam | Coef. Std. Err. z P>|z| [95% Conf. Interval]

-------------+----------------------------------------------------------------

overall |

group_1 | .0049986 .0011701 4.27 0.000 .0027053 .0072919

group_2 | .0155417 .0026955 5.77 0.000 .0102586 .0208249

difference | -.0105431 .0029385 -3.59 0.000 -.0163026 -.0047837

explained | -.001551 .0014607 -1.06 0.288 -.0044139 .001312

unexplained | -.0089922 .0035448 -2.54 0.011 -.0159398 -.0020445

-------------+----------------------------------------------------------------

explained |

mage2 | .0001428 .0001359 1.05 0.293 -.0001236 .0004092

mage3 | -.000075 .0000867 -0.87 0.387 -.000245 .000095

medu1 | -.0002754 .0005009 -0.55 0.583 -.0012572 .0007065

medu2 | .0001309 .0001211 1.08 0.280 -.0001064 .0003683

rural1 | .0007618 .0008384 0.91 0.364 -.0008814 .002405

notwork | .0001984 .0002411 0.82 0.411 -.0002741 .0006709

media | -.0012013 .0007388 -1.63 0.104 -.0026493 .0002466

water | -.0000155 .0001082 -0.14 0.886 -.0002275 .0001966

toilety | .0001066 .0003403 0.31 0.754 -.0005603 .0007736

mar1 | .0003039 .0001445 2.10 0.035 .0000208 .0005871

mar2 | 9.77e-06 .0000279 0.35 0.726 -.0000449 .0000644

sex | .0000278 .0000308 0.90 0.367 -.0000326 .0000881

age | .0000179 .0000347 0.52 0.607 -.0000502 .0000859

weightm2 | -.0000407 .0000505 -0.81 0.420 -.0001396 .0000582

weightm3 | -.0000419 .0000376 -1.11 0.265 -.0001156 .0000318

border1 | -.0000188 .0000334 -0.56 0.574 -.0000843 .0000467

border2 | 3.07e-06 .0000168 0.18 0.855 -.0000298 .000036

border3 | -.0000106 .000021 -0.51 0.613 -.0000519 .0000306

bint2 | -3.38e-06 .0000118 -0.29 0.774 -.0000265 .0000197

bint3 | 0 (omitted)

com_zsesc52 | .0003501 .00037 0.95 0.344 -.000375 .0010753

com_zsesc53 | 5.58e-06 .0000565 0.10 0.921 -.0001052 .0001164

com_zsesc54 | -.0004822 .0004979 -0.97 0.333 -.0014582 .0004937

com_zsesc55 | -.0014447 .0013706 -1.05 0.292 -.0041309 .0012416

-------------+----------------------------------------------------------------

unexplained |

mage2 | -.0014902 .00625 -0.24 0.812 -.01374 .0107596

mage3 | .0000517 .0036023 0.01 0.989 -.0070087 .0071121

medu1 | -.0679133 .0298645 -2.27 0.023 -.1264466 -.00938

medu2 | -.0411781 .0181981 -2.26 0.024 -.0768458 -.0055105

rural1 | -.0130475 .0098494 -1.32 0.185 -.032352 .006257

notwork | .0002944 .000254 1.16 0.247 -.0002035 .0007922

media | -.0006003 .0020381 -0.29 0.768 -.0045949 .0033942

water | .0017907 .0051359 0.35 0.727 -.0082755 .0118569

toilety | -.0051148 .0035098 -1.46 0.145 -.0119939 .0017643

mar1 | -.0002435 .0002231 -1.09 0.275 -.0006808 .0001938

mar2 | -.0063248 .0085334 -0.74 0.459 -.0230499 .0104004

sex | .0007232 .0022841 0.32 0.752 -.0037535 .0051999

age | -.005401 .0037249 -1.45 0.147 -.0127017 .0018996

weightm2 | -.0002788 .0007574 -0.37 0.713 -.0017632 .0012057

weightm3 | -.0001828 .0004019 -0.45 0.649 -.0009706 .0006049

border1 | .000042 .000076 0.55 0.581 -.000107 .0001909

border2 | -.0000331 .0013658 -0.02 0.981 -.0027101 .0026438

border3 | -.0003574 .0012603 -0.28 0.777 -.0028275 .0021127

bint2 | -.0043336 .0063565 -0.68 0.495 -.0167922 .0081249

bint3 | -.0042164 .0052567 -0.80 0.422 -.0145193 .0060865

com_zsesc52 | .006494 .0032135 2.02 0.043 .0001957 .0127924

com_zsesc53 | .0080368 .0035575 2.26 0.024 .0010642 .0150093

com_zsesc54 | .0047216 .0030305 1.56 0.119 -.0012181 .0106614

com_zsesc55 | .0069352 .0030639 2.26 0.024 .0009301 .0129403

_cons | .112634 .0588416 1.91 0.056 -.0026935 .2279615

------------------------------------------------------------------------------

4

(border1 dropped from model 1)

(border1 dropped from model 2)

(border1 dropped from pooled model)

(border1 missing in model 1; assumed zero)

(border1 missing in model 2; assumed zero)

(border1 missing in pooled model; assumed zero)

Blinder-Oaxaca decomposition Number of obs = 7805

Model = logit

Group 1: poor = 0 N of obs 1 = 3914

Group 2: poor = 1 N of obs 2 = 3891

------------------------------------------------------------------------------

| Robust

sam | Coef. Std. Err. z P>|z| [95% Conf. Interval]

-------------+----------------------------------------------------------------

overall |

group_1 | .0242718 .0024565 9.88 0.000 .0194573 .0290864

group_2 | .0321254 .0028153 11.41 0.000 .0266076 .0376432

difference | -.0078536 .0037363 -2.10 0.036 -.0151766 -.0005306

explained | -.0009685 .0022792 -0.42 0.671 -.0054356 .0034987

unexplained | -.0068851 .0046591 -1.48 0.139 -.0160169 .0022466

-------------+----------------------------------------------------------------

explained |

mage2 | -.0000591 .0000945 -0.63 0.532 -.0002444 .0001261

mage3 | .0000537 .0000827 0.65 0.516 -.0001083 .0002157

medu1 | -.0006419 .0009275 -0.69 0.489 -.0024597 .0011759

medu2 | -.0003338 .000468 -0.71 0.476 -.0012511 .0005834

rural1 | -.0047709 .0045761 -1.04 0.297 -.01374 .0041982

notwork | .0006324 .0006208 1.02 0.308 -.0005844 .0018491

media | .0000825 .0006405 0.13 0.897 -.0011729 .0013379

water | .0018758 .0013892 1.35 0.177 -.0008471 .0045986

toilety | .0002601 .0003697 0.70 0.482 -.0004645 .0009846

mar1 | .0000382 .0001332 0.29 0.774 -.0002229 .0002994

mar2 | -3.15e-07 .0000637 -0.00 0.996 -.0001252 .0001246

sex | 3.82e-06 .0000646 0.06 0.953 -.0001228 .0001304

age | -6.97e-06 .0000859 -0.08 0.935 -.0001753 .0001614

weightm2 | -5.87e-06 .0000173 -0.34 0.734 -.0000397 .000028

weightm3 | 6.09e-06 .0000266 0.23 0.819 -.0000461 .0000583

border1 | 0 (omitted)

border2 | -2.87e-06 .0000187 -0.15 0.878 -.0000395 .0000337

border3 | 7.52e-07 9.77e-06 0.08 0.939 -.0000184 .0000199

bint2 | .0000611 .0001093 0.56 0.576 -.0001532 .0002754

bint3 | -2.22e-06 .0000371 -0.06 0.952 -.000075 .0000706

com_zsesc52 | .0000147 .0004296 0.03 0.973 -.0008274 .0008568

com_zsesc53 | .0000377 .000246 0.15 0.878 -.0004446 .0005199

com_zsesc54 | .0005534 .0008857 0.62 0.532 -.0011825 .0022893

com_zsesc55 | .0012353 .0016864 0.73 0.464 -.00207 .0045406

-------------+----------------------------------------------------------------

unexplained |

mage2 | -.0111184 .0111209 -1.00 0.317 -.032915 .0106783

mage3 | -.0098159 .0075716 -1.30 0.195 -.0246559 .0050241

medu1 | .0063156 .0048897 1.29 0.196 -.0032681 .0158993

medu2 | -.0011774 .0065044 -0.18 0.856 -.0139257 .0115709

rural1 | .0154827 .0160223 0.97 0.334 -.0159203 .0468858

notwork | -.0034357 .0035475 -0.97 0.333 -.0103886 .0035172

media | .0031951 .0040981 0.78 0.436 -.004837 .0112272

water | -.0083134 .0073164 -1.14 0.256 -.0226534 .0060265

toilety | -.0049975 .0054139 -0.92 0.356 -.0156085 .0056135

mar1 | -.0013191 .0015269 -0.86 0.388 -.0043118 .0016736

mar2 | -.0198474 .0213423 -0.93 0.352 -.0616776 .0219827

sex | -.0053279 .0069581 -0.77 0.444 -.0189655 .0083097

age | .0073633 .011392 0.65 0.518 -.0149647 .0296912

weightm2 | .0026504 .0021579 1.23 0.219 -.0015791 .0068798

weightm3 | .0001906 .0007366 0.26 0.796 -.0012532 .0016344

border1 | 0 (omitted)

border2 | -.0040679 .0038976 -1.04 0.297 -.011707 .0035711

border3 | -.0022117 .0028452 -0.78 0.437 -.0077883 .0033648

bint2 | .0065368 .0124057 0.53 0.598 -.0177779 .0308515

bint3 | .0096853 .0088009 1.10 0.271 -.0075642 .0269349

com_zsesc52 | .0018455 .0050448 0.37 0.715 -.0080421 .011733

com_zsesc53 | -.0007162 .0062649 -0.11 0.909 -.0129951 .0115627

com_zsesc54 | .0005679 .0061989 0.09 0.927 -.0115817 .0127175

com_zsesc55 | .0035231 .0070139 0.50 0.615 -.0102239 .0172701

_cons | .0081073 .0342024 0.24 0.813 -.0589282 .0751429

------------------------------------------------------------------------------

5

(bint3 com_zsesc55 dropped from model 1)

(bint3 dropped from model 2)

(model 2 has zero variance coefficients)

(border1 dropped from pooled model)

(bint3 com_zsesc55 missing in model 1; assumed zero)

(bint3 missing in model 2; assumed zero)

(border1 missing in pooled model; assumed zero)

Blinder-Oaxaca decomposition Number of obs = 4760

Model = logit

Group 1: poor = 0 N of obs 1 = 2666

Group 2: poor = 1 N of obs 2 = 2094

------------------------------------------------------------------------------

| Robust

sam | Coef. Std. Err. z P>|z| [95% Conf. Interval]

-------------+----------------------------------------------------------------

overall |

group_1 | .007877 .0017118 4.60 0.000 .004522 .011232

group_2 | .0267431 .0039872 6.71 0.000 .0189283 .0345579

difference | -.0188661 .0043391 -4.35 0.000 -.0273706 -.0103616

explained | -.0182481 .0102911 -1.77 0.076 -.0384182 .001922

unexplained | -.000618 .0118032 -0.05 0.958 -.0237518 .0225157

-------------+----------------------------------------------------------------

explained |

mage2 | 6.84e-06 .0001209 0.06 0.955 -.0002302 .0002439

mage3 | .000246 .0002545 0.97 0.334 -.0002528 .0007447

medu1 | -.0013284 .0025112 -0.53 0.597 -.0062502 .0035934

medu2 | .0010256 .0006832 1.50 0.133 -.0003135 .0023646

rural1 | .0048555 .0098321 0.49 0.621 -.0144149 .024126

notwork | .000546 .00049 1.11 0.265 -.0004143 .0015063

media | -.0021551 .003912 -0.55 0.582 -.0098224 .0055122

water | .0028028 .0021123 1.33 0.185 -.0013373 .0069429

toilety | -.004675 .0034583 -1.35 0.176 -.0114531 .0021031

mar1 | .0003367 .0003425 0.98 0.326 -.0003346 .0010079

mar2 | -.000314 .0003949 -0.80 0.427 -.001088 .00046

sex | .0000356 .0002628 0.14 0.892 -.0004794 .0005506

age | -.0000434 .0001096 -0.40 0.692 -.0002583 .0001715

weightm2 | -.0000965 .000119 -0.81 0.417 -.0003297 .0001367

weightm3 | -.0011914 .000445 -2.68 0.007 -.0020636 -.0003193

border1 | 0 (omitted)

border2 | .000555 .000441 1.26 0.208 -.0003094 .0014195

border3 | -.0001191 .0002891 -0.41 0.680 -.0006857 .0004476

bint2 | -.0008861 .00092 -0.96 0.335 -.0026893 .000917

bint3 | .0002967 .000307 0.97 0.334 -.0003049 .0008984

com_zsesc52 | .0003524 .0028663 0.12 0.902 -.0052654 .0059701

com_zsesc53 | .0002427 .0003813 0.64 0.524 -.0005047 .0009901

com_zsesc54 | -.0051525 .0049627 -1.04 0.299 -.0148792 .0045742

com_zsesc55 | -.0135883 .0091136 -1.49 0.136 -.0314507 .0042741

-------------+----------------------------------------------------------------

unexplained |

mage2 | .0000499 .0010602 0.05 0.962 -.002028 .0021278

mage3 | .0001588 .0031936 0.05 0.960 -.0061006 .0064182

medu1 | -.0001751 .0034812 -0.05 0.960 -.0069981 .0066479

medu2 | -.0002339 .0046814 -0.05 0.960 -.0094092 .0089414

rural1 | -.000214 .0044603 -0.05 0.962 -.008956 .008528

notwork | -.0000993 .0019515 -0.05 0.959 -.0039242 .0037255

media | .0004289 .0085993 0.05 0.960 -.0164255 .0172833

water | -.0001741 .0035601 -0.05 0.961 -.0071518 .0068037

toilety | .0003464 .0069376 0.05 0.960 -.0132511 .0139438

mar1 | .0009921 .0197341 0.05 0.960 -.0376861 .0396703

mar2 | .0151001 .3004643 0.05 0.960 -.5737991 .6039993

sex | .0002917 .0058094 0.05 0.960 -.0110945 .0116779

age | .0003864 .0077111 0.05 0.960 -.014727 .0154998

weightm2 | -.0000402 .0007933 -0.05 0.960 -.0015951 .0015146

weightm3 | .0000274 .0005421 0.05 0.960 -.0010352 .00109

border1 | -.0002341 .0046396 -0.05 0.960 -.0093276 .0088594

border2 | .000109 .0021678 0.05 0.960 -.0041399 .0043579

border3 | .0002683 .0053398 0.05 0.960 -.0101974 .0107341

bint2 | -2.75e-06 .0002973 -0.01 0.993 -.0005855 .00058

bint3 | .0000164 .0003286 0.05 0.960 -.0006276 .0006604

com_zsesc52 | .0005393 .0107472 0.05 0.960 -.0205248 .0216033

com_zsesc53 | .0022243 .044271 0.05 0.960 -.0845453 .0889938

com_zsesc54 | .0046907 .0933148 0.05 0.960 -.1782029 .1875843

com_zsesc55 | .0056618 .1126309 0.05 0.960 -.2150908 .2264144

_cons | -.0307359 .6111111 -0.05 0.960 -1.228492 1.16702

------------------------------------------------------------------------------

6

(mar1 border1 dropped from model 1)

(mar1 bint3 dropped from model 2)

(mar1 bint3 dropped from pooled model)

(mar1 border1 missing in model 1; assumed zero)

(mar1 bint3 missing in model 2; assumed zero)

(mar1 bint3 missing in pooled model; assumed zero)

Blinder-Oaxaca decomposition Number of obs = 8688

Model = logit

Group 1: poor = 0 N of obs 1 = 3995

Group 2: poor = 1 N of obs 2 = 4693

------------------------------------------------------------------------------

| Robust

sam | Coef. Std. Err. z P>|z| [95% Conf. Interval]

-------------+----------------------------------------------------------------

overall |

group_1 | .02403 .0024173 9.94 0.000 .0192922 .0287678

group_2 | .0432559 .0029591 14.62 0.000 .0374562 .0490556

difference | -.0192259 .0038209 -5.03 0.000 -.0267148 -.011737

explained | -.0119151 .0037664 -3.16 0.002 -.019297 -.0045332

unexplained | -.0073108 .0059037 -1.24 0.216 -.0188818 .0042602

-------------+----------------------------------------------------------------

explained |

mage2 | -.0003356 .0001906 -1.76 0.078 -.0007092 .000038

mage3 | .0001138 .0001179 0.97 0.334 -.0001172 .0003449

medu1 | -.0026708 .0026792 -1.00 0.319 -.007922 .0025803

medu2 | -.0003501 .0012663 -0.28 0.782 -.002832 .0021318

rural1 | .0054809 .0033584 1.63 0.103 -.0011013 .0120632

notwork | -.0001999 .0004107 -0.49 0.626 -.0010048 .000605

media | -.0026756 .0019817 -1.35 0.177 -.0065597 .0012085

water | .0016072 .0011216 1.43 0.152 -.0005912 .0038055

toilety | .0010137 .0013909 0.73 0.466 -.0017124 .0037397

mar1 | 0 (omitted)

mar2 | .0000618 .0000647 0.95 0.340 -.0000651 .0001886

sex | -.0000302 .0000562 -0.54 0.591 -.0001403 .00008

age | .0003742 .000209 1.79 0.073 -.0000353 .0007838

weightm2 | -.000443 .0001983 -2.23 0.025 -.0008317 -.0000543

weightm3 | -.0009603 .0003022 -3.18 0.001 -.0015525 -.000368

border1 | -.0008209 .0005774 -1.42 0.155 -.0019525 .0003107

border2 | -.000839 .0003771 -2.22 0.026 -.0015782 -.0000999

border3 | -.0000176 .0000398 -0.44 0.658 -.0000957 .0000604

bint2 | -.000321 .0006665 -0.48 0.630 -.0016272 .0009852

bint3 | 0 (omitted)

com_zsesc52 | .0016472 .0016402 1.00 0.315 -.0015674 .0048619

com_zsesc53 | -.0000569 .0001437 -0.40 0.692 -.0003386 .0002247

com_zsesc54 | -.0042431 .0021529 -1.97 0.049 -.0084627 -.0000234

com_zsesc55 | -.0082498 .004199 -1.96 0.049 -.0164797 -.0000199

-------------+----------------------------------------------------------------

unexplained |

mage2 | -.0030191 .0054078 -0.56 0.577 -.0136181 .00758

mage3 | .001215 .0031116 0.39 0.696 -.0048837 .0073136

medu1 | .0107695 .0108136 1.00 0.319 -.0104248 .0319637

medu2 | .0050747 .0045088 1.13 0.260 -.0037623 .0139118

rural1 | -.003144 .0173401 -0.18 0.856 -.03713 .0308421

notwork | .0045053 .0044948 1.00 0.316 -.0043044 .0133149

media | -.0003484 .0023705 -0.15 0.883 -.0049944 .0042977

water | .0046506 .0058064 0.80 0.423 -.0067297 .016031

toilety | -.0006564 .0017996 -0.36 0.715 -.0041837 .0028708

mar1 | 0 (omitted)

mar2 | -.0125183 .0135437 -0.92 0.355 -.0390634 .0140268

sex | .0023182 .0034679 0.67 0.504 -.0044788 .0091152

age | .0012406 .0059217 0.21 0.834 -.0103657 .0128469

weightm2 | -.0004583 .001012 -0.45 0.651 -.0024419 .0015252

weightm3 | -.0006886 .0014008 -0.49 0.623 -.0034342 .002057

border1 | .0013412 .0017943 0.75 0.455 -.0021756 .004858

border2 | -.0012853 .0022205 -0.58 0.563 -.0056374 .0030668

border3 | -.0032699 .0019086 -1.71 0.087 -.0070107 .0004709

bint2 | .008639 .0048341 1.79 0.074 -.0008357 .0181137

bint3 | .0081256 .0050841 1.60 0.110 -.0018389 .0180902

com_zsesc52 | -.0003854 .0039262 -0.10 0.922 -.0080807 .0073098

com_zsesc53 | .0004447 .0048048 0.09 0.926 -.0089724 .0098619

com_zsesc54 | .0016105 .0050452 0.32 0.750 -.0082779 .0114989

com_zsesc55 | .0008184 .0053173 0.15 0.878 -.0096033 .0112401

_cons | -.0322905 .0314992 -1.03 0.305 -.0940278 .0294468

------------------------------------------------------------------------------

7

(water mar1 weightm3 border1 border3 bint3 com_zsesc54 com_zsesc55 dropped from model 1)

(model 1 has zero variance coefficients)

(weightm3 bint2 dropped from model 2)

(model 2 has zero variance coefficients)

(weightm3 border1 dropped from pooled model)

(water mar1 weightm3 border1 border3 bint3 com_zsesc54 com_zsesc55 missing in model 1; assumed zero)

(weightm3 bint2 missing in model 2; assumed zero)

(weightm3 border1 missing in pooled model; assumed zero)

Blinder-Oaxaca decomposition Number of obs = 1929

Model = logit

Group 1: poor = 0 N of obs 1 = 571

Group 2: poor = 1 N of obs 2 = 1358

------------------------------------------------------------------------------

| Robust

sam | Coef. Std. Err. z P>|z| [95% Conf. Interval]

-------------+----------------------------------------------------------------

overall |

group_1 | .0105079 .0044484 2.36 0.018 .0017891 .0192267

group_2 | .0125184 .0033355 3.75 0.000 .005981 .0190558

difference | -.0020105 .00556 -0.36 0.718 -.012908 .0088869

explained | .0025964 .0045578 0.57 0.569 -.0063368 .0115296

unexplained | -.0046069 .0064677 -0.71 0.476 -.0172834 .0080696

-------------+----------------------------------------------------------------

explained |

mage2 | .0002075 .0003501 0.59 0.553 -.0004787 .0008938

mage3 | .0000658 .0001898 0.35 0.729 -.0003063 .0004379

medu1 | -.0001868 .0008983 -0.21 0.835 -.0019474 .0015738

medu2 | -.0000294 .0000667 -0.44 0.659 -.0001602 .0001014

rural1 | .0031116 .003634 0.86 0.392 -.004011 .0102341

notwork | .0001573 .0001939 0.81 0.417 -.0002227 .0005373

media | .0000232 .0003914 0.06 0.953 -.0007439 .0007904

water | .0011489 .0013692 0.84 0.401 -.0015346 .0038325

toilety | -.0002923 .0009401 -0.31 0.756 -.0021347 .0015502

mar1 | .0002109 .0003946 0.53 0.593 -.0005624 .0009843

mar2 | .0000635 .0001958 0.32 0.746 -.0003202 .0004473

sex | .000039 .0000743 0.52 0.600 -.0001067 .0001847

age | .0001552 .0002162 0.72 0.473 -.0002685 .0005789

weightm2 | -.0001132 .0001581 -0.72 0.474 -.0004231 .0001967

weightm3 | 0 (omitted)

border1 | 0 (omitted)

border2 | -.0009268 .0012245 -0.76 0.449 -.0033267 .0014732

border3 | .000608 .0009672 0.63 0.530 -.0012877 .0025036

bint2 | -.0001465 .000204 -0.72 0.473 -.0005464 .0002534

bint3 | -.0007747 .0007878 -0.98 0.325 -.0023188 .0007695

com_zsesc52 | .0004123 .0009402 0.44 0.661 -.0014304 .002255

com_zsesc53 | .0002254 .0002796 0.81 0.420 -.0003226 .0007733

com_zsesc54 | .0009347 .0019131 0.49 0.625 -.002815 .0046844

com_zsesc55 | -.0022974 .0022944 -1.00 0.317 -.0067942 .0021995

-------------+----------------------------------------------------------------

unexplained |

mage2 | .0318428 .0298843 1.07 0.287 -.0267292 .0904149

mage3 | .0229801 .0215766 1.07 0.287 -.0193094 .0652695

medu1 | .0011224 .0014717 0.76 0.446 -.001762 .0040068

medu2 | .0017329 .0017894 0.97 0.333 -.0017743 .0052402

rural1 | -.0002103 .0022473 -0.09 0.925 -.0046151 .0041944

notwork | .0002237 .0007816 0.29 0.775 -.0013083 .0017556

media | .0023233 .0033107 0.70 0.483 -.0041655 .0088121

water | -.0044966 .0052658 -0.85 0.393 -.0148173 .0058241

toilety | -.000052 .0023023 -0.02 0.982 -.0045644 .0044605

mar1 | -.0028359 .0026545 -1.07 0.285 -.0080387 .0023669

mar2 | -.0609107 .0574684 -1.06 0.289 -.1735467 .0517252

sex | .0036933 .0044823 0.82 0.410 -.0050919 .0124784

age | .0018695 .0038383 0.49 0.626 -.0056534 .0093924

weightm2 | -.0002221 .0006712 -0.33 0.741 -.0015377 .0010936

weightm3 | 0 (omitted)

border1 | -.0005849 .0008439 -0.69 0.488 -.002239 .0010692

border2 | -.0001412 .0015954 -0.09 0.929 -.0032681 .0029857

border3 | -.0001004 .0001709 -0.59 0.557 -.0004353 .0002345

bint2 | .0017297 .0019233 0.90 0.368 -.0020398 .0054992

bint3 | .0014922 .0020789 0.72 0.473 -.0025824 .0055668

com_zsesc52 | -.0053968 .0049011 -1.10 0.271 -.0150028 .0042092

com_zsesc53 | -.0109152 .0101243 -1.08 0.281 -.0307585 .0089281

com_zsesc54 | -.0190778 .0177284 -1.08 0.282 -.0538249 .0156692

com_zsesc55 | -.019702 .0183235 -1.08 0.282 -.0556155 .0162115

_cons | .0510291 .0507094 1.01 0.314 -.0483595 .1504176

------------------------------------------------------------------------------

8

(bint2 dropped from model 1)

(model 2 has zero variance coefficients)

(pooled model has zero variance coefficients)

(bint2 missing in model 1; assumed zero)

Blinder-Oaxaca decomposition Number of obs = 208386

Model = logit

Group 1: poor = 0 N of obs 1 = 105851

Group 2: poor = 1 N of obs 2 = 102535

------------------------------------------------------------------------------

| Robust

sam | Coef. Std. Err. z P>|z| [95% Conf. Interval]

-------------+----------------------------------------------------------------

overall |

group_1 | .0648931 .0007565 85.78 0.000 .0634103 .0663759

group_2 | .0839128 .0008653 96.97 0.000 .0822168 .0856088

difference | -.0190197 .0011494 -16.55 0.000 -.0212725 -.0167669

explained | -.012624 .0012513 -10.09 0.000 -.0150765 -.0101714

unexplained | -.0063958 .0017282 -3.70 0.000 -.0097829 -.0030086

-------------+----------------------------------------------------------------

explained |

mage2 | -.000359 .0000735 -4.88 0.000 -.0005032 -.0002149

mage3 | .0004133 .0000945 4.37 0.000 .000228 .0005985

medu1 | -.0024174 .0005874 -4.12 0.000 -.0035688 -.0012661

medu2 | -.0001287 .0001296 -0.99 0.321 -.0003828 .0001254

rural1 | .0022862 .0008314 2.75 0.006 .0006566 .0039158

notwork | -.000017 .000048 -0.35 0.723 -.000111 .000077

media | -.0007707 .0007382 -1.04 0.296 -.0022175 .0006762

water | -.0002543 .000123 -2.07 0.039 -.0004954 -.0000133

toilety | -.0088055 .0008917 -9.87 0.000 -.0105532 -.0070577

mar1 | 1.89e-07 3.05e-06 0.06 0.951 -5.79e-06 6.17e-06

mar2 | -1.38e-06 2.74e-06 -0.50 0.616 -6.75e-06 4.00e-06

sex | .0001337 .0000275 4.87 0.000 .0000799 .0001876

age | -.0000436 .0000694 -0.63 0.530 -.0001796 .0000924

weightm2 | -.0000941 .0000306 -3.07 0.002 -.0001541 -.000034

weightm3 | -.0001591 .0000295 -5.39 0.000 -.000217 -.0001013

border1 | .098832 .0019729 50.09 0.000 .0949652 .1026989

border2 | -.0002008 .0001199 -1.67 0.094 -.0004359 .0000343

border3 | -1.63e-06 .0001036 -0.02 0.987 -.0002047 .0002015

bint2 | -.0880481 .0019045 -46.23 0.000 -.0917808 -.0843153

bint3 | -.0111469 .001365 -8.17 0.000 -.0138222 -.0084717

com_zsesc52 | .0000277 .0005198 0.05 0.957 -.000991 .0010464

com_zsesc53 | -4.65e-06 .000011 -0.42 0.672 -.0000262 .0000169

com_zsesc54 | .0001217 .0005872 0.21 0.836 -.0010293 .0012726

com_zsesc55 | -.001986 .0011404 -1.74 0.082 -.004221 .0002491

-------------+----------------------------------------------------------------

unexplained |

mage2 | .0007539 .0017836 0.42 0.673 -.0027419 .0042497

mage3 | -.0002689 .0005443 -0.49 0.621 -.0013356 .0007978

medu1 | .0012977 .0009752 1.33 0.183 -.0006137 .0032091

medu2 | .0003979 .0005488 0.73 0.468 -.0006776 .0014735

rural1 | -.0063443 .0045506 -1.39 0.163 -.0152634 .0025747

notwork | .0007305 .0004833 1.51 0.131 -.0002167 .0016776

media | .0066821 .0030592 2.18 0.029 .0006862 .0126781

water | .011516 .0038587 2.98 0.003 .003953 .0190789

toilety | .0058248 .0017313 3.36 0.001 .0024315 .009218

mar1 | .0000147 .0000517 0.28 0.777 -.0000868 .0001161

mar2 | .0396782 .0120988 3.28 0.001 .0159651 .0633914

sex | .0007867 .0012771 0.62 0.538 -.0017164 .0032898

age | -.0030622 .0022269 -1.38 0.169 -.0074268 .0013025

weightm2 | -.0003355 .0003794 -0.88 0.376 -.0010791 .0004081

weightm3 | .0001655 .0001809 0.92 0.360 -.000189 .0005201

border1 | -.3449591 .0175387 -19.67 0.000 -.3793343 -.310584

border2 | .0055349 .0015157 3.65 0.000 .0025642 .0085055

border3 | .0006872 .0007496 0.92 0.359 -.000782 .0021564

bint2 | -.2582023 .0131412 -19.65 0.000 -.2839587 -.232446

bint3 | -.2142901 .010934 -19.60 0.000 -.2357204 -.1928598

com_zsesc52 | .0015406 .0011827 1.30 0.193 -.0007775 .0038587

com_zsesc53 | .002639 .0014934 1.77 0.077 -.000288 .005566

com_zsesc54 | .0011994 .001745 0.69 0.492 -.0022207 .0046195

com_zsesc55 | .0018169 .0019192 0.95 0.344 -.0019446 .0055784

_cons | .7398006 .0395053 18.73 0.000 .6623716 .8172296

------------------------------------------------------------------------------

9

(border1 dropped from model 1)

(bint3 dropped from model 2)

(model 2 has zero variance coefficients)

(border1 dropped from pooled model)

(border1 missing in model 1; assumed zero)

(bint3 missing in model 2; assumed zero)

(border1 missing in pooled model; assumed zero)

Blinder-Oaxaca decomposition Number of obs = 8721

Model = logit

Group 1: poor = 0 N of obs 1 = 3895

Group 2: poor = 1 N of obs 2 = 4826

------------------------------------------------------------------------------

| Robust

sam | Coef. Std. Err. z P>|z| [95% Conf. Interval]

-------------+----------------------------------------------------------------

overall |

group_1 | .0092426 .0015071 6.13 0.000 .0062888 .0121965

group_2 | .0140903 .0018861 7.47 0.000 .0103936 .0177871

difference | -.0048477 .0024143 -2.01 0.045 -.0095797 -.0001158

explained | -.0141468 .0066908 -2.11 0.034 -.0272606 -.001033

unexplained | .0092991 .0077689 1.20 0.231 -.0059277 .0245258

-------------+----------------------------------------------------------------

explained |

mage2 | .0002328 .0004335 0.54 0.591 -.0006169 .0010825

mage3 | -.0002008 .0003325 -0.60 0.546 -.0008524 .0004509

medu1 | -.0014283 .0023475 -0.61 0.543 -.0060292 .0031727

medu2 | -.0001052 .0003422 -0.31 0.759 -.0007759 .0005656

rural1 | .0021286 .0018914 1.13 0.260 -.0015784 .0058356

notwork | -.0004948 .0004868 -1.02 0.309 -.0014488 .0004592

media | -.0058919 .0029532 -2.00 0.046 -.0116801 -.0001038

water | .0014787 .0014125 1.05 0.295 -.0012897 .004247

toilety | -.0016464 .0024584 -0.67 0.503 -.0064648 .0031719

mar1 | -.0002152 .0002303 -0.93 0.350 -.0006665 .0002362

mar2 | .000066 .0000814 0.81 0.417 -.0000934 .0002255

sex | .0001296 .0001082 1.20 0.231 -.0000825 .0003417

age | 7.31e-06 .0000528 0.14 0.890 -.0000962 .0001108

weightm2 | 1.70e-06 .0000329 0.05 0.959 -.0000628 .0000662

weightm3 | -.0001047 .0000875 -1.20 0.231 -.0002761 .0000668

border1 | 0 (omitted)

border2 | .0001108 .0004515 0.25 0.806 -.0007741 .0009958

border3 | -.0000791 .0001001 -0.79 0.429 -.0002752 .0001171

bint2 | .000252 .001456 0.17 0.863 -.0026018 .0031057

bint3 | .0004605 .0006093 0.76 0.450 -.0007337 .0016547

com_zsesc52 | -.0002471 .0009129 -0.27 0.787 -.0020363 .0015422

com_zsesc53 | .0001201 .0003476 0.35 0.730 -.0005612 .0008014

com_zsesc54 | -.0011537 .0008168 -1.41 0.158 -.0027546 .0004471

com_zsesc55 | -.0075678 .0040214 -1.88 0.060 -.0154496 .0003139

-------------+----------------------------------------------------------------

unexplained |

mage2 | .0055365 .0043332 1.28 0.201 -.0029564 .0140294

mage3 | .0018686 .0018749 1.00 0.319 -.0018062 .0055433

medu1 | -.0011166 .0019992 -0.56 0.576 -.0050349 .0028018

medu2 | .0004906 .0036994 0.13 0.894 -.00676 .0077413

rural1 | -.001229 .0030755 -0.40 0.689 -.0072568 .0047988

notwork | .0002595 .0015926 0.16 0.871 -.002862 .003381

media | -.0021962 .0035413 -0.62 0.535 -.0091369 .0047446

water | .001966 .0031592 0.62 0.534 -.004226 .008158

toilety | -.0052305 .0035906 -1.46 0.145 -.0122681 .001807

mar1 | .0000533 .0007301 0.07 0.942 -.0013777 .0014842

mar2 | .0023155 .005328 0.43 0.664 -.0081272 .0127582

sex | -.0003695 .0017749 -0.21 0.835 -.0038482 .0031093

age | -.0124231 .0086783 -1.43 0.152 -.0294323 .0045861

weightm2 | -.0000135 .0006783 -0.02 0.984 -.001343 .001316

weightm3 | .0000744 .0002229 0.33 0.739 -.0003625 .0005113

border1 | .000351 .0007024 0.50 0.617 -.0010257 .0017277

border2 | -.0015126 .0015207 -0.99 0.320 -.0044932 .0014679

border3 | .0006826 .0009453 0.72 0.470 -.0011701 .0025354

bint2 | .0048322 .0034281 1.41 0.159 -.0018868 .0115511

bint3 | .0004704 .0019094 0.25 0.805 -.003272 .0042128

com_zsesc52 | -.0202207 .0120488 -1.68 0.093 -.0438359 .0033945

com_zsesc53 | -.0249291 .0146392 -1.70 0.089 -.0536214 .0037633

com_zsesc54 | -.034059 .0199537 -1.71 0.088 -.0731675 .0050495

com_zsesc55 | -.045628 .0265795 -1.72 0.086 -.0977229 .006467

_cons | .1393261 .085955 1.62 0.105 -.0291427 .3077948

------------------------------------------------------------------------------

10

(mage3 medu1 media water toilety mar2 sex age weightm2 weightm3 border1 border3 bint2 bint3 com_zsesc53 com_zsesc54 com_zsesc55 dropped from model 1)

(model 1 has zero variance coefficients)

(medu1 rural1 mar1 weightm3 border3 bint3 com_zsesc52 dropped from model 2)

(model 2 has zero variance coefficients)

(medu1 weightm3 border3 bint3 dropped from pooled model)

(pooled model has zero variance coefficients)

(mage3 medu1 media water toilety mar2 sex age weightm2 weightm3 border1 border3 bint2 bint3 com_zsesc53 com_zsesc54 com_zsesc55 missing in model 1; assu

> med zero)

(medu1 rural1 mar1 weightm3 border3 bint3 com_zsesc52 missing in model 2; assumed zero)

(medu1 weightm3 border3 bint3 missing in pooled model; assumed zero)

Blinder-Oaxaca decomposition Number of obs = 393

Model = logit

Group 1: poor = 0 N of obs 1 = 20

Group 2: poor = 1 N of obs 2 = 373

------------------------------------------------------------------------------

| Robust

sam | Coef. Std. Err. z P>|z| [95% Conf. Interval]

-------------+----------------------------------------------------------------

overall |

group_1 | .1 5.85e-08 1.7e+06 0.000 .0999999 .1000001

group_2 | .0294906 .008253 3.57 0.000 .0133151 .0456662

difference | .0705094 .008253 8.54 0.000 .0543338 .0866849

explained | -.898777 .0548331 -16.39 0.000 -1.006248 -.7913062

unexplained | .9692864 .0554409 17.48 0.000 .8606241 1.077949

-------------+----------------------------------------------------------------

explained |

mage2 | -.0320585 .0225836 -1.42 0.156 -.0763215 .0122046

mage3 | -.0019014 .0187206 -0.10 0.919 -.0385931 .0347903

medu1 | 0 (omitted)

medu2 | -.0923408 .0311867 -2.96 0.003 -.1534657 -.0312159

rural1 | -.08098 .0704606 -1.15 0.250 -.2190803 .0571202

notwork | -.046581 .0291103 -1.60 0.110 -.1036362 .0104741

media | .003795 .0277987 0.14 0.891 -.0506894 .0582794

water | .0406983 .040853 1.00 0.319 -.0393721 .1207687

toilety | .0604421 .0317483 1.90 0.057 -.0017835 .1226676

mar1 | -.0269709 .0259234 -1.04 0.298 -.0777799 .023838

mar2 | -.0059338 .0596946 -0.10 0.921 -.1229331 .1110654

sex | -.0494881 .0228115 -2.17 0.030 -.0941978 -.0047783

age | -.0305178 .0097595 -3.13 0.002 -.0496461 -.0113894

weightm2 | -.0180916 .0082091 -2.20 0.028 -.0341811 -.0020021

weightm3 | 0 (omitted)

border1 | -.0018895 .0304871 -0.06 0.951 -.061643 .057864

border2 | .0070773 .0144146 0.49 0.623 -.0211748 .0353295

border3 | 0 (omitted)

bint2 | -.009244 .016381 -0.56 0.573 -.0413501 .0228622

bint3 | 0 (omitted)

com_zsesc52 | -.053237 .0497184 -1.07 0.284 -.1506833 .0442092

com_zsesc53 | -.1377211 .0347651 -3.96 0.000 -.2058595 -.0695828

com_zsesc54 | -.1650225 .0423827 -3.89 0.000 -.248091 -.0819539

com_zsesc55 | -.2588117 .0711337 -3.64 0.000 -.3982312 -.1193921

-------------+----------------------------------------------------------------

unexplained |

mage2 | 4.624386 3.972802 1.16 0.244 -3.162162 12.41093

mage3 | .0011836 .00383 0.31 0.757 -.0063231 .0086903

medu1 | 0 (omitted)

medu2 | -.8454658 1.044317 -0.81 0.418 -2.892289 1.201357

rural1 | -1.714901 1.733693 -0.99 0.323 -5.112877 1.683075

notwork | .0348885 .055511 0.63 0.530 -.0739111 .143688

media | .0080011 .0798718 0.10 0.920 -.1485447 .1645469

water | .2086129 .2793011 0.75 0.455 -.3388072 .7560331

toilety | .164424 .1899965 0.87 0.387 -.2079624 .5368103

mar1 | -.0594672 .0745112 -0.80 0.425 -.2055065 .0865721

mar2 | .0023172 .0145646 0.16 0.874 -.0262288 .0308632

sex | -.0153501 .0217025 -0.71 0.479 -.0578863 .027186

age | -.2602652 .2630221 -0.99 0.322 -.7757791 .2552487

weightm2 | -.0035212 .0044566 -0.79 0.429 -.0122561 .0052136

weightm3 | 0 (omitted)

border1 | .0006634 .131055 0.01 0.996 -.2561996 .2575265

border2 | -2.707602 2.838767 -0.95 0.340 -8.271483 2.856279

border3 | 0 (omitted)

bint2 | .0001215 .0053989 0.02 0.982 -.0104602 .0107032

bint3 | 0 (omitted)

com_zsesc52 | 1.427454 1.383764 1.03 0.302 -1.284674 4.139582

com_zsesc53 | .0146663 .0260566 0.56 0.574 -.0364038 .0657363

com_zsesc54 | .0275416 .0372482 0.74 0.460 -.0454636 .1005469

com_zsesc55 | .0444685 .0585777 0.76 0.448 -.0703418 .1592787

_cons | .0171307 .3796901 0.05 0.964 -.7270484 .7613097

------------------------------------------------------------------------------

11

(border1 dropped from model 1)

(model 1 has zero variance coefficients)

(rural1 mar1 bint3 com_zsesc53 dropped from model 2)

(bint3 dropped from pooled model)

(border1 missing in model 1; assumed zero)

(rural1 mar1 bint3 com_zsesc53 missing in model 2; assumed zero)

(bint3 missing in pooled model; assumed zero)

Blinder-Oaxaca decomposition Number of obs = 4119

Model = logit

Group 1: poor = 0 N of obs 1 = 2470

Group 2: poor = 1 N of obs 2 = 1649

------------------------------------------------------------------------------

| Robust

sam | Coef. Std. Err. z P>|z| [95% Conf. Interval]

-------------+----------------------------------------------------------------

overall |

group_1 | .0392713 .0040589 9.68 0.000 .031316 .0472265

group_2 | .0654942 .0060866 10.76 0.000 .0535647 .0774238

difference | -.026223 .0073158 -3.58 0.000 -.0405617 -.0118842

explained | -.0109703 .0068203 -1.61 0.108 -.0243378 .0023973

unexplained | -.0152527 .0105205 -1.45 0.147 -.0358726 .0053672

-------------+----------------------------------------------------------------

explained |

mage2 | -.0000114 .0000571 -0.20 0.841 -.0001234 .0001005

mage3 | .0001179 .0002941 0.40 0.689 -.0004585 .0006943

medu1 | -.0012869 .0025678 -0.50 0.616 -.0063197 .0037458

medu2 | .001073 .0009101 1.18 0.238 -.0007107 .0028567

rural1 | .0153548 .0052398 2.93 0.003 .0050849 .0256247

notwork | -.0003198 .0002806 -1.14 0.254 -.0008698 .0002302

media | -.0026552 .0017621 -1.51 0.132 -.0061088 .0007984

water | -.0034553 .0024145 -1.43 0.152 -.0081876 .001277

toilety | -.0101063 .0046402 -2.18 0.029 -.0192009 -.0010116

mar1 | .00026 .000508 0.51 0.609 -.0007356 .0012556

mar2 | -2.10e-06 .0003054 -0.01 0.995 -.0006007 .0005965

sex | -7.41e-06 .0000361 -0.20 0.838 -.0000782 .0000634

age | .0003545 .000327 1.08 0.278 -.0002865 .0009955

weightm2 | -.0000243 .0000634 -0.38 0.701 -.0001487 .0001

weightm3 | .0000898 .0001374 0.65 0.513 -.0001795 .000359

border1 | .000088 .0002898 0.30 0.761 -.00048 .0006559

border2 | .0002858 .0003025 0.94 0.345 -.0003071 .0008786

border3 | -.0000791 .0001527 -0.52 0.604 -.0003785 .0002202

bint2 | .0000261 .0003955 0.07 0.947 -.000749 .0008012

bint3 | 0 (omitted)

com_zsesc52 | .0053492 .002575 2.08 0.038 .0003022 .0103961

com_zsesc53 | -.0005725 .0005793 -0.99 0.323 -.0017079 .0005629

com_zsesc54 | -.0051051 .0032019 -1.59 0.111 -.0113808 .0011705

com_zsesc55 | -.0103438 .0062314 -1.66 0.097 -.0225572 .0018696

-------------+----------------------------------------------------------------

unexplained |

mage2 | .0066557 .0075912 0.88 0.381 -.0082227 .0215342

mage3 | .0000875 .0044 0.02 0.984 -.0085363 .0087113

medu1 | .0629546 .0350116 1.80 0.072 -.0056669 .1315761

medu2 | .0024611 .0026058 0.94 0.345 -.0026462 .0075684

rural1 | -.0354147 .0162557 -2.18 0.029 -.0672752 -.0035542

notwork | -.005807 .0060902 -0.95 0.340 -.0177436 .0061297

media | .007801 .0064801 1.20 0.229 -.0048998 .0205018

water | -.0020027 .007914 -0.25 0.800 -.0175138 .0135085

toilety | -.0019895 .0041335 -0.48 0.630 -.0100909 .006112

mar1 | .0080325 .0026098 3.08 0.002 .0029175 .0131476

mar2 | .616674 .1692381 3.64 0.000 .2849734 .9483745

sex | .0011213 .0053176 0.21 0.833 -.0093011 .0115437

age | -.0070544 .0096496 -0.73 0.465 -.0259673 .0118584

weightm2 | .0006742 .0015266 0.44 0.659 -.0023179 .0036662

weightm3 | .0014847 .0013658 1.09 0.277 -.0011922 .0041616

border1 | .0012055 .002514 0.48 0.632 -.0037219 .0061329

border2 | -.0018606 .0027528 -0.68 0.499 -.007256 .0035349

border3 | -.0052139 .0032084 -1.63 0.104 -.0115022 .0010745

bint2 | -.0015609 .0072527 -0.22 0.830 -.0157759 .0126541

bint3 | -.0053981 .0056398 -0.96 0.338 -.0164518 .0056556

com_zsesc52 | .0011481 .0017348 0.66 0.508 -.002252 .0045483

com_zsesc53 | .0006582 .0042081 0.16 0.876 -.0075894 .0089059

com_zsesc54 | .0096848 .0062614 1.55 0.122 -.0025873 .0219568

com_zsesc55 | .0100175 .0077091 1.30 0.194 -.0050921 .025127

_cons | -.6796118 .2001403 -3.40 0.001 -1.07188 -.287344

------------------------------------------------------------------------------

12

(weightm3 bint2 dropped from model 1)

(weightm3 bint3 dropped from model 2)

(model 2 has zero variance coefficients)

(weightm3 border1 dropped from pooled model)

(weightm3 bint2 missing in model 1; assumed zero)

(weightm3 bint3 missing in model 2; assumed zero)

(weightm3 border1 missing in pooled model; assumed zero)

Blinder-Oaxaca decomposition Number of obs = 8765

Model = logit

Group 1: poor = 0 N of obs 1 = 5489

Group 2: poor = 1 N of obs 2 = 3276

------------------------------------------------------------------------------

| Robust

sam | Coef. Std. Err. z P>|z| [95% Conf. Interval]

-------------+----------------------------------------------------------------

overall |

group_1 | .012024 .0014702 8.18 0.000 .0091426 .0149055

group_2 | .0247253 .0027387 9.03 0.000 .0193575 .030093

difference | -.0127012 .0031083 -4.09 0.000 -.0187935 -.006609

explained | -.0078191 .0027791 -2.81 0.005 -.013266 -.0023722

unexplained | -.0048822 .0045183 -1.08 0.280 -.0137379 .0039736

-------------+----------------------------------------------------------------

explained |

mage2 | -8.98e-07 .0000511 -0.02 0.986 -.0001011 .0000993

mage3 | -.0001259 .0003961 -0.32 0.751 -.0009023 .0006505

medu1 | -.0024227 .0016904 -1.43 0.152 -.0057358 .0008903

medu2 | .0006928 .0004047 1.71 0.087 -.0001004 .001486

rural1 | .0028634 .0013974 2.05 0.040 .0001245 .0056023

notwork | -.000175 .0001653 -1.06 0.290 -.0004989 .0001489

media | -.0011498 .0005292 -2.17 0.030 -.0021869 -.0001126

water | .0005874 .001178 0.50 0.618 -.0017214 .0028963

toilety | .0000322 .0009121 0.04 0.972 -.0017554 .0018198

mar1 | .0000533 .0003485 0.15 0.879 -.0006297 .0007362

mar2 | .0000625 .0000946 0.66 0.509 -.0001228 .0002478

sex | .0000642 .0000564 1.14 0.255 -.0000463 .0001747

age | .0000776 .0000741 1.05 0.295 -.0000676 .0002227

weightm2 | -.0001021 .0000663 -1.54 0.123 -.0002319 .0000278

weightm3 | 0 (omitted)

border1 | 0 (omitted)

border2 | -.0000191 .0001724 -0.11 0.912 -.000357 .0003188

border3 | 3.62e-06 .0000872 0.04 0.967 -.0001672 .0001745

bint2 | -.0002226 .0004579 -0.49 0.627 -.0011202 .0006749

bint3 | .0000806 .0001856 0.43 0.664 -.0002832 .0004443

com_zsesc52 | .0013046 .0011437 1.14 0.254 -.0009371 .0035463

com_zsesc53 | .0003378 .0001975 1.71 0.087 -.0000492 .0007248

com_zsesc54 | -.0028196 .0012232 -2.31 0.021 -.0052171 -.0004221

com_zsesc55 | -.0069411 .0029913 -2.32 0.020 -.012804 -.0010783

-------------+----------------------------------------------------------------

unexplained |

mage2 | .0048793 .0049997 0.98 0.329 -.0049199 .0146786

mage3 | .0006868 .0029193 0.24 0.814 -.0050349 .0064084

medu1 | .006862 .0117475 0.58 0.559 -.0161627 .0298866

medu2 | .0081001 .0122642 0.66 0.509 -.0159372 .0321374

rural1 | -.0106957 .0092199 -1.16 0.246 -.0287663 .0073749

notwork | .0011044 .0037501 0.29 0.768 -.0062457 .0084545

media | -.0036753 .0033297 -1.10 0.270 -.0102013 .0028508

water | .0045128 .0046671 0.97 0.334 -.0046346 .0136602

toilety | .0003958 .0014272 0.28 0.782 -.0024014 .003193

mar1 | .0003529 .0009445 0.37 0.709 -.0014983 .0022041

mar2 | -.0011848 .0092972 -0.13 0.899 -.019407 .0170375

sex | -.0007139 .0035999 -0.20 0.843 -.0077695 .0063418

age | .0071106 .0081706 0.87 0.384 -.0089034 .0231247

weightm2 | -.0012272 .0012991 -0.94 0.345 -.0037735 .001319

weightm3 | 0 (omitted)

border1 | -.0047372 .0035959 -1.32 0.188 -.011785 .0023107

border2 | -.0011264 .0020825 -0.54 0.589 -.0052079 .0029552

border3 | -.0007897 .0017895 -0.44 0.659 -.0042971 .0027177

bint2 | -.0006668 .0026118 -0.26 0.798 -.0057858 .0044522

bint3 | .0006628 .0023282 0.28 0.776 -.0039005 .0052261

com_zsesc52 | -.0192296 .009187 -2.09 0.036 -.0372358 -.0012234

com_zsesc53 | -.0586061 .0286602 -2.04 0.041 -.114779 -.0024331

com_zsesc54 | -.0946729 .0467172 -2.03 0.043 -.186237 -.0031088

com_zsesc55 | -.1320212 .0647723 -2.04 0.042 -.2589726 -.0050699

_cons | .289797 .1433634 2.02 0.043 .0088099 .5707841

------------------------------------------------------------------------------

13

(mage3 weightm2 weightm3 bint2 com_zsesc54 dropped from model 1)

(bint2 com_zsesc52 dropped from model 2)

(bint2 dropped from pooled model)

(mage3 weightm2 weightm3 bint2 com_zsesc54 missing in model 1; assumed zero)

(bint2 com_zsesc52 missing in model 2; assumed zero)

(bint2 missing in pooled model; assumed zero)

Blinder-Oaxaca decomposition Number of obs = 1107

Model = logit

Group 1: poor = 0 N of obs 1 = 464

Group 2: poor = 1 N of obs 2 = 643

------------------------------------------------------------------------------

| Robust

sam | Coef. Std. Err. z P>|z| [95% Conf. Interval]

-------------+----------------------------------------------------------------

overall |

group_1 | .0258621 .0071471 3.62 0.000 .011854 .0398702

group_2 | .0342146 .0068397 5.00 0.000 .020809 .0476203

difference | -.0083526 .0098926 -0.84 0.398 -.0277417 .0110366

explained | -.0676103 .0418546 -1.62 0.106 -.1496438 .0144232

unexplained | .0592577 .0453164 1.31 0.191 -.0295607 .1480762

-------------+----------------------------------------------------------------

explained |

mage2 | .004477 .0081239 0.55 0.582 -.0114455 .0203995

mage3 | -.0261968 .0171475 -1.53 0.127 -.0598052 .0074116

medu1 | -.0069539 .0039954 -1.74 0.082 -.0147848 .0008769

medu2 | -.0060981 .0065964 -0.92 0.355 -.0190269 .0068306

rural1 | .0049998 .0213685 0.23 0.815 -.0368818 .0468814

notwork | .0037898 .0056054 0.68 0.499 -.0071965 .0147761

media | -.0137825 .0092573 -1.49 0.137 -.0319264 .0043614

water | .0029993 .0081214 0.37 0.712 -.0129184 .0189169

toilety | .0031739 .020344 0.16 0.876 -.0366996 .0430475

mar1 | -.0012485 .0038921 -0.32 0.748 -.008877 .0063799

mar2 | .0035888 .0032884 1.09 0.275 -.0028563 .0100339

sex | .001181 .0017875 0.66 0.509 -.0023225 .0046845

age | .0042189 .0026844 1.57 0.116 -.0010424 .0094801

weightm2 | -.0088818 .0068669 -1.29 0.196 -.0223406 .0045771

weightm3 | -.004753 .0029808 -1.59 0.111 -.0105952 .0010893

border1 | .0003744 .0087843 0.04 0.966 -.0168426 .0175914

border2 | -.000387 .0035755 -0.11 0.914 -.0073948 .0066207

border3 | .0002282 .0005908 0.39 0.699 -.0009298 .0013863

bint2 | 0 (omitted)

bint3 | -.0001214 .0005113 -0.24 0.812 -.0011236 .0008807

com_zsesc52 | -.0198076 .0157113 -1.26 0.207 -.0506013 .010986

com_zsesc53 | -.0000195 .0019641 -0.01 0.992 -.003869 .0038301

com_zsesc54 | -.0124711 .0219106 -0.57 0.569 -.0554151 .0304728

com_zsesc55 | .0040799 .0229444 0.18 0.859 -.0408904 .0490501

-------------+----------------------------------------------------------------

unexplained |

mage2 | .0014945 .0665545 0.02 0.982 -.1289499 .1319389

mage3 | .0062165 .0269909 0.23 0.818 -.0466846 .0591176

medu1 | -.0072501 .0100145 -0.72 0.469 -.0268783 .012378

medu2 | -.0242438 .0368757 -0.66 0.511 -.0965188 .0480312

rural1 | -.1494969 .1758406 -0.85 0.395 -.4941381 .1951443

notwork | .0004719 .0472382 0.01 0.992 -.0921133 .0930571

media | -.0910169 .1541487 -0.59 0.555 -.3931427 .2111089

water | -.196779 .2237299 -0.88 0.379 -.6352815 .2417236

toilety | -.0040668 .0259544 -0.16 0.875 -.0549365 .0468029

mar1 | .0102063 .0751606 0.14 0.892 -.1371057 .1575184

mar2 | .0448179 .0954178 0.47 0.639 -.1421975 .2318333

sex | .0890301 .1225496 0.73 0.468 -.1511627 .3292229

age | -.0338999 .1033857 -0.33 0.743 -.2365322 .1687325

weightm2 | -.0015907 .0025751 -0.62 0.537 -.0066378 .0034563

weightm3 | .0013917 .001895 0.73 0.463 -.0023224 .0051058

border1 | -.010712 .0461495 -0.23 0.816 -.1011634 .0797393

border2 | .0294408 .0408658 0.72 0.471 -.0506547 .1095362

border3 | -.0159837 .024695 -0.65 0.517 -.064385 .0324177

bint2 | 0 (omitted)

bint3 | -.0191133 .0427468 -0.45 0.655 -.1028954 .0646688

com_zsesc52 | .0087809 .0144994 0.61 0.545 -.0196375 .0371992

com_zsesc53 | .0706784 .0742175 0.95 0.341 -.0747852 .2161419

com_zsesc54 | .0641401 .0715235 0.90 0.370 -.0760435 .2043237

com_zsesc55 | .1037206 .118702 0.87 0.382 -.1289311 .3363722

_cons | .1830212 .3589934 0.51 0.610 -.5205929 .8866353

------------------------------------------------------------------------------

14

(border1 dropped from model 1)

(rural1 toilety mar1 border1 com_zsesc52 dropped from model 2)

(border1 dropped from pooled model)

(border1 missing in model 1; assumed zero)

(rural1 toilety mar1 border1 com_zsesc52 missing in model 2; assumed zero)

(border1 missing in pooled model; assumed zero)

Blinder-Oaxaca decomposition Number of obs = 4597

Model = logit

Group 1: poor = 0 N of obs 1 = 2992

Group 2: poor = 1 N of obs 2 = 1605

------------------------------------------------------------------------------

| Robust

sam | Coef. Std. Err. z P>|z| [95% Conf. Interval]

-------------+----------------------------------------------------------------

overall |

group_1 | .0601604 .0043332 13.88 0.000 .0516674 .0686534

group_2 | .0785047 .0067108 11.70 0.000 .0653518 .0916575

difference | -.0183442 .0079882 -2.30 0.022 -.0340008 -.0026877

explained | -.0116481 .0055304 -2.11 0.035 -.0224874 -.0008088

unexplained | -.0066962 .0097533 -0.69 0.492 -.0258124 .01242

-------------+----------------------------------------------------------------

explained |

mage2 | .0000503 .00025 0.20 0.841 -.0004398 .0005403

mage3 | .0000326 .0002901 0.11 0.911 -.0005359 .0006011

medu1 | .0035429 .002608 1.36 0.174 -.0015686 .0086544

medu2 | -.0022603 .0016245 -1.39 0.164 -.0054443 .0009237

rural1 | .0174866 .0060178 2.91 0.004 .0056919 .0292813

notwork | .0012067 .0009649 1.25 0.211 -.0006845 .0030979

media | -.0063534 .0021052 -3.02 0.003 -.0104794 -.0022273

water | -.0026183 .002499 -1.05 0.295 -.0075163 .0022797

toilety | .0062939 .0036747 1.71 0.087 -.0009085 .0134963

mar1 | .0001966 .0001971 1.00 0.318 -.0001897 .0005829

mar2 | .0001053 .0001447 0.73 0.467 -.0001783 .0003889

sex | -.0000537 .0002706 -0.20 0.843 -.0005841 .0004768

age | -.0003259 .0003098 -1.05 0.293 -.0009331 .0002813

weightm2 | -.0004065 .0005473 -0.74 0.458 -.0014792 .0006662

weightm3 | -.0002231 .0003136 -0.71 0.477 -.0008378 .0003916

border1 | 0 (omitted)

border2 | -.0007708 .000496 -1.55 0.120 -.001743 .0002014

border3 | .0002336 .0002434 0.96 0.337 -.0002434 .0007107

bint2 | -.0001292 .0010215 -0.13 0.899 -.0021312 .0018729

bint3 | .0001828 .0007945 0.23 0.818 -.0013744 .00174

com_zsesc52 | .0083414 .0021137 3.95 0.000 .0041986 .0124842

com_zsesc53 | -.0009018 .0007201 -1.25 0.210 -.0023132 .0005095

com_zsesc54 | -.0107805 .0028148 -3.83 0.000 -.0162974 -.0052635

com_zsesc55 | -.0244974 .0066095 -3.71 0.000 -.0374518 -.011543

-------------+----------------------------------------------------------------

unexplained |

mage2 | -.0297834 .0424359 -0.70 0.483 -.1129563 .0533895

mage3 | -.0062532 .0151055 -0.41 0.679 -.0358594 .0233531

medu1 | .1467809 .2118559 0.69 0.488 -.268449 .5620108

medu2 | .0031984 .0080908 0.40 0.693 -.0126593 .0190562

rural1 | -.0951584 .1221126 -0.78 0.436 -.3344947 .144178

notwork | .0107723 .0284122 0.38 0.705 -.0449146 .0664592

media | .0082742 .0159107 0.52 0.603 -.0229101 .0394585

water | .0488361 .0659879 0.74 0.459 -.0804977 .17817

toilety | -.0008688 .0017401 -0.50 0.618 -.0042793 .0025418

mar1 | .000219 .0003638 0.60 0.547 -.0004941 .000932

mar2 | .1030524 .1537807 0.67 0.503 -.1983522 .4044571

sex | .0043528 .0137042 0.32 0.751 -.0225069 .0312124

age | -.0155814 .026964 -0.58 0.563 -.0684298 .0372669

weightm2 | .0116444 .0156745 0.74 0.458 -.0190771 .0423659

weightm3 | .0034751 .0055208 0.63 0.529 -.0073454 .0142957

border1 | 0 (omitted)

border2 | .000071 .0068297 0.01 0.992 -.013315 .013457

border3 | .0006473 .0065009 0.10 0.921 -.0120942 .0133887

bint2 | .0171667 .0384083 0.45 0.655 -.0581122 .0924456

bint3 | -.0048463 .0190325 -0.25 0.799 -.0421492 .0324567

com_zsesc52 | .017149 .0219721 0.78 0.435 -.0259155 .0602135

com_zsesc53 | .0210455 .0312335 0.67 0.500 -.040171 .0822619

com_zsesc54 | .0272735 .0354776 0.77 0.442 -.0422614 .0968083

com_zsesc55 | .0405351 .052739 0.77 0.442 -.0628314 .1439017

_cons | -.3186985 .4334977 -0.74 0.462 -1.168338 .5309413

------------------------------------------------------------------------------

15

(bint3 dropped from model 1)

(border1 dropped from model 2)

(bint3 dropped from pooled model)

(bint3 missing in model 1; assumed zero)

(border1 missing in model 2; assumed zero)

(bint3 missing in pooled model; assumed zero)

Blinder-Oaxaca decomposition Number of obs = 23785

Model = logit

Group 1: poor = 0 N of obs 1 = 13447

Group 2: poor = 1 N of obs 2 = 10338

------------------------------------------------------------------------------

| Robust

sam | Coef. Std. Err. z P>|z| [95% Conf. Interval]

-------------+----------------------------------------------------------------

overall |

group_1 | .0620213 .0020684 29.99 0.000 .0579673 .0660752

group_2 | .0925711 .002853 32.45 0.000 .0869794 .0981628

difference | -.0305498 .0035239 -8.67 0.000 -.0374565 -.0236432

explained | -.0238695 .0045997 -5.19 0.000 -.0328848 -.0148543

unexplained | -.0066803 .0060341 -1.11 0.268 -.0185069 .0051463

-------------+----------------------------------------------------------------

explained |

mage2 | -.0006094 .000337 -1.81 0.071 -.00127 .0000512

mage3 | .0001483 .0001062 1.40 0.162 -.0000598 .0003564

medu1 | -.0119632 .0030833 -3.88 0.000 -.0180064 -.0059199

medu2 | .0006583 .0003824 1.72 0.085 -.0000912 .0014078

rural1 | .0184923 .0023044 8.02 0.000 .0139758 .0230088

notwork | .00019 .0003385 0.56 0.575 -.0004735 .0008534

media | .0017523 .0016184 1.08 0.279 -.0014197 .0049242

water | .0038323 .0012651 3.03 0.002 .0013529 .0063118

toilety | .0081317 .0011174 7.28 0.000 .0059417 .0103217

mar1 | .0001969 .0001913 1.03 0.303 -.000178 .0005718

mar2 | -.0000143 .0001924 -0.07 0.941 -.0003914 .0003628

sex | .0001171 .000078 1.50 0.133 -.0000358 .0002701

age | .000079 .0001517 0.52 0.603 -.0002183 .0003762

weightm2 | -.0006195 .0002101 -2.95 0.003 -.0010313 -.0002077

weightm3 | -.0008739 .000187 -4.67 0.000 -.0012405 -.0005074

border1 | -.0009038 .0004246 -2.13 0.033 -.001736 -.0000716

border2 | -.0004361 .0002403 -1.81 0.070 -.0009071 .0000349

border3 | -.0001158 .0001239 -0.93 0.350 -.0003585 .000127

bint2 | .0001682 .0002641 0.64 0.524 -.0003494 .0006857

bint3 | 0 (omitted)

com_zsesc52 | .0109426 .0013902 7.87 0.000 .0082178 .0136674

com_zsesc53 | .0041427 .0005759 7.19 0.000 .0030139 .0052715

com_zsesc54 | -.0215114 .0025883 -8.31 0.000 -.0265844 -.0164384

com_zsesc55 | -.0356739 .00458 -7.79 0.000 -.0446506 -.0266972

-------------+----------------------------------------------------------------

unexplained |

mage2 | -.0025807 .0035633 -0.72 0.469 -.0095647 .0044033

mage3 | -.004696 .0029747 -1.58 0.114 -.0105264 .0011344

medu1 | -.0049366 .0066419 -0.74 0.457 -.0179544 .0080813

medu2 | -.0017726 .0019815 -0.89 0.371 -.0056563 .0021112

rural1 | -.0176472 .0094985 -1.86 0.063 -.0362639 .0009695

notwork | -.0021374 .0016414 -1.30 0.193 -.0053545 .0010797

media | .0002497 .002371 0.11 0.916 -.0043974 .0048968

water | .0035148 .0035317 1.00 0.320 -.0034072 .0104367

toilety | -.0009709 .0025725 -0.38 0.706 -.0060129 .0040711

mar1 | -.000894 .0005651 -1.58 0.114 -.0020016 .0002137

mar2 | -.0273436 .0200632 -1.36 0.173 -.0666667 .0119796

sex | .0008264 .0022459 0.37 0.713 -.0035754 .0052282

age | -.0084425 .0049222 -1.72 0.086 -.0180897 .0012048

weightm2 | -.0000932 .000703 -0.13 0.894 -.0014712 .0012847

weightm3 | .0002385 .00034 0.70 0.483 -.0004279 .0009049

border1 | -.0033265 .0017828 -1.87 0.062 -.0068207 .0001678

border2 | -.0019074 .0014872 -1.28 0.200 -.0048222 .0010075

border3 | -.0021335 .0013889 -1.54 0.125 -.0048558 .0005887

bint2 | -.0026191 .0039294 -0.67 0.505 -.0103206 .0050825

bint3 | -.0003988 .0020735 -0.19 0.847 -.0044628 .0036652

com_zsesc52 | .0016051 .0018468 0.87 0.385 -.0020146 .0052247

com_zsesc53 | .0006775 .0029658 0.23 0.819 -.0051353 .0064903

com_zsesc54 | .0050967 .0057481 0.89 0.375 -.0061693 .0163627

com_zsesc55 | .0058728 .0068342 0.86 0.390 -.0075221 .0192676

_cons | .0571381 .0344381 1.66 0.097 -.0103593 .1246356

------------------------------------------------------------------------------

16

(toilety mar1 bint3 com_zsesc55 dropped from model 1)

(mar1 mar2 border1 dropped from model 2)

(mar1 bint3 dropped from pooled model)

(toilety mar1 bint3 com_zsesc55 missing in model 1; assumed zero)

(mar1 mar2 border1 missing in model 2; assumed zero)

(mar1 bint3 missing in pooled model; assumed zero)

Blinder-Oaxaca decomposition Number of obs = 3854

Model = logit

Group 1: poor = 0 N of obs 1 = 1995

Group 2: poor = 1 N of obs 2 = 1859

------------------------------------------------------------------------------

| Robust

sam | Coef. Std. Err. z P>|z| [95% Conf. Interval]

-------------+----------------------------------------------------------------

overall |

group_1 | .0210526 .0032173 6.54 0.000 .0147469 .0273583

group_2 | .0462614 .0048335 9.57 0.000 .0367879 .055735

difference | -.0252088 .0058064 -4.34 0.000 -.036589 -.0138286

explained | -.0056654 .0045567 -1.24 0.214 -.0145964 .0032655

unexplained | -.0195434 .0078594 -2.49 0.013 -.0349474 -.0041393

-------------+----------------------------------------------------------------

explained |

mage2 | -.0001354 .0004507 -0.30 0.764 -.0010188 .000748

mage3 | .000242 .000501 0.48 0.629 -.0007399 .001224

medu1 | -.0027158 .0044011 -0.62 0.537 -.0113418 .0059103

medu2 | .0000848 .0002872 0.30 0.768 -.0004782 .0006478

rural1 | .0138346 .0047098 2.94 0.003 .0046035 .0230657

notwork | .00118 .0007743 1.52 0.128 -.0003376 .0026976

media | -.0043883 .0029845 -1.47 0.141 -.0102379 .0014613

water | -.0002886 .0009421 -0.31 0.759 -.0021351 .0015579

toilety | .0051178 .0034521 1.48 0.138 -.0016481 .0118838

mar1 | 0 (omitted)

mar2 | .000261 .0004299 0.61 0.544 -.0005815 .0011035

sex | -8.36e-06 .0001008 -0.08 0.934 -.000206 .0001893

age | -.0000215 .0003522 -0.06 0.951 -.0007118 .0006688

weightm2 | .0000803 .0001573 0.51 0.610 -.000228 .0003887

weightm3 | .0001645 .0002939 0.56 0.576 -.0004114 .0007405

border1 | -.0001738 .0007624 -0.23 0.820 -.0016681 .0013205

border2 | .0004914 .0005987 0.82 0.412 -.0006821 .0016649

border3 | .0000222 .0001664 0.13 0.894 -.000304 .0003483

bint2 | .0007196 .0004563 1.58 0.115 -.0001748 .001614

bint3 | 0 (omitted)

com_zsesc52 | .0002785 .0023699 0.12 0.906 -.0043663 .0049233

com_zsesc53 | .0006101 .0004215 1.45 0.148 -.0002161 .0014362

com_zsesc54 | -.0064707 .0027894 -2.32 0.020 -.0119378 -.0010037

com_zsesc55 | -.0145499 .0062998 -2.31 0.021 -.0268973 -.0022024

-------------+----------------------------------------------------------------

unexplained |

mage2 | .0077966 .009959 0.78 0.434 -.0117226 .0273159

mage3 | .0056263 .0045844 1.23 0.220 -.003359 .0146115

medu1 | .0104829 .0112276 0.93 0.350 -.0115227 .0324885

medu2 | .0023687 .0031112 0.76 0.446 -.0037292 .0084666

rural1 | -.0020205 .0083232 -0.24 0.808 -.0183336 .0142927

notwork | -.0115468 .0211889 -0.54 0.586 -.0530762 .0299826

media | .0143349 .010008 1.43 0.152 -.0052805 .0339503

water | .028801 .0325318 0.89 0.376 -.0349602 .0925621

toilety | -.0150225 .0086179 -1.74 0.081 -.0319132 .0018682

mar1 | 0 (omitted)

mar2 | -.0237093 .0301142 -0.79 0.431 -.0827321 .0353135

sex | -.0041766 .0066239 -0.63 0.528 -.0171592 .0088061

age | -.0027735 .0103544 -0.27 0.789 -.0230678 .0175207

weightm2 | .0030894 .0025601 1.21 0.228 -.0019284 .0081071

weightm3 | .0002828 .001278 0.22 0.825 -.002222 .0027875

border1 | .0029584 .0051241 0.58 0.564 -.0070846 .0130014

border2 | -.0052503 .0041945 -1.25 0.211 -.0134714 .0029709

border3 | -.0066597 .0039058 -1.71 0.088 -.014315 .0009956

bint2 | .022798 .0102437 2.23 0.026 .0027207 .0428753

bint3 | -.0021567 .003996 -0.54 0.589 -.0099887 .0056754

com_zsesc52 | -.0053757 .0042269 -1.27 0.203 -.0136603 .0029089

com_zsesc53 | -.002761 .0058075 -0.48 0.634 -.0141434 .0086215

com_zsesc54 | -.0005784 .0077935 -0.07 0.941 -.0158534 .0146965

com_zsesc55 | -.0045939 .009304 -0.49 0.621 -.0228293 .0136416

_cons | -.0314576 .0590451 -0.53 0.594 -.1471839 .0842687

------------------------------------------------------------------------------

17

(bint2 dropped from model 1)

(border1 dropped from model 2)

(model 2 has zero variance coefficients)

(bint3 dropped from pooled model)

(bint2 missing in model 1; assumed zero)

(border1 missing in model 2; assumed zero)

(bint3 missing in pooled model; assumed zero)

Blinder-Oaxaca decomposition Number of obs = 10392

Model = logit

Group 1: poor = 0 N of obs 1 = 4500

Group 2: poor = 1 N of obs 2 = 5892

------------------------------------------------------------------------------

| Robust

sam | Coef. Std. Err. z P>|z| [95% Conf. Interval]

-------------+----------------------------------------------------------------

overall |

group_1 | .0113333 .0015762 7.19 0.000 .008244 .0144227

group_2 | .0198574 .0019177 10.35 0.000 .0160989 .023616

difference | -.0085241 .0024823 -3.43 0.001 -.0133894 -.0036588

explained | -.0030343 .0023069 -1.32 0.188 -.0075557 .001487

unexplained | -.0054898 .0034257 -1.60 0.109 -.012204 .0012244

-------------+----------------------------------------------------------------

explained |

mage2 | .000084 .0001023 0.82 0.412 -.0001165 .0002845

mage3 | .0000379 .0000457 0.83 0.407 -.0000518 .0001276

medu1 | -.0009108 .0009297 -0.98 0.327 -.0027331 .0009114

medu2 | .0002803 .000474 0.59 0.554 -.0006487 .0012093

rural1 | .0042877 .0016791 2.55 0.011 .0009967 .0075788

notwork | .0001357 .0001532 0.89 0.376 -.0001645 .0004359

media | -.0004994 .0007002 -0.71 0.476 -.0018717 .0008729

water | .0001162 .0004226 0.28 0.783 -.0007121 .0009445

toilety | -.0004191 .0011582 -0.36 0.717 -.0026891 .0018508

mar1 | .0000296 .0000921 0.32 0.747 -.0001508 .0002101

mar2 | -.0000607 .0001512 -0.40 0.688 -.000357 .0002357

sex | 5.32e-06 .0000478 0.11 0.911 -.0000884 .0000991

age | 3.91e-06 .0000383 0.10 0.919 -.0000711 .0000789

weightm2 | .0001337 .0000748 1.79 0.074 -.0000129 .0002802

weightm3 | -.0002596 .0001053 -2.46 0.014 -.000466 -.0000532

border1 | -.0000849 .0002344 -0.36 0.717 -.0005443 .0003746

border2 | .0000216 .0001333 0.16 0.872 -.0002397 .0002829

border3 | 3.89e-06 .0000633 0.06 0.951 -.0001201 .0001279

bint2 | .0002896 .0002898 1.00 0.318 -.0002783 .0008576

bint3 | 0 (omitted)

com_zsesc52 | .001638 .0005968 2.74 0.006 .0004683 .0028076

com_zsesc53 | .0000638 .0001255 0.51 0.611 -.0001822 .0003097

com_zsesc54 | -.0031696 .0012329 -2.57 0.010 -.005586 -.0007532

com_zsesc55 | -.0047615 .0018594 -2.56 0.010 -.0084059 -.0011171

-------------+----------------------------------------------------------------

unexplained |

mage2 | -.0128132 .0277658 -0.46 0.644 -.0672332 .0416067

mage3 | -.0158458 .0325514 -0.49 0.626 -.0796454 .0479538

medu1 | -.0030825 .0243407 -0.13 0.899 -.0507894 .0446245

medu2 | -.0046346 .0120665 -0.38 0.701 -.0282845 .0190153

rural1 | -.0334041 .0618206 -0.54 0.589 -.1545703 .087762

notwork | .0047602 .0117629 0.40 0.686 -.0182946 .0278151

media | -.0016222 .0185678 -0.09 0.930 -.0380144 .0347699

water | -.0089764 .0256252 -0.35 0.726 -.0592008 .0412481

toilety | .0210549 .0416586 0.51 0.613 -.0605946 .1027043

mar1 | .0000875 .001938 0.05 0.964 -.003711 .0038859

mar2 | -.0166563 .063048 -0.26 0.792 -.1402281 .1069155

sex | -.0249272 .0486042 -0.51 0.608 -.1201897 .0703354

age | .0013391 .0169359 0.08 0.937 -.0318546 .0345327

weightm2 | .0035491 .0073307 0.48 0.628 -.0108189 .0179171

weightm3 | .001756 .004083 0.43 0.667 -.0062466 .0097585

border1 | .0058666 .0111151 0.53 0.598 -.0159187 .0276518

border2 | -.0024411 .0076376 -0.32 0.749 -.0174106 .0125283

border3 | -.0000997 .0046445 -0.02 0.983 -.0092028 .0090033

bint2 | -.002594 .0108197 -0.24 0.811 -.0238002 .0186122

bint3 | .0132468 .0261469 0.51 0.612 -.0380002 .0644938

com_zsesc52 | -.0893376 .1723186 -0.52 0.604 -.4270758 .2484006

com_zsesc53 | -.1504829 .2929322 -0.51 0.607 -.7246194 .4236536

com_zsesc54 | -.2458417 .4741584 -0.52 0.604 -1.175175 .6834917

com_zsesc55 | -.2658865 .5131788 -0.52 0.604 -1.271699 .7399256

_cons | .8214959 1.618579 0.51 0.612 -2.35086 3.993852

------------------------------------------------------------------------------

18

(bint3 dropped from model 1)

(mar1 border1 dropped from model 2)

(bint3 dropped from pooled model)

(bint3 missing in model 1; assumed zero)

(mar1 border1 missing in model 2; assumed zero)

(bint3 missing in pooled model; assumed zero)

Blinder-Oaxaca decomposition Number of obs = 4626

Model = logit

Group 1: poor = 0 N of obs 1 = 3006

Group 2: poor = 1 N of obs 2 = 1620

------------------------------------------------------------------------------

| Robust

sam | Coef. Std. Err. z P>|z| [95% Conf. Interval]

-------------+----------------------------------------------------------------

overall |

group_1 | .0874917 .005152 16.98 0.000 .077394 .0975894

group_2 | .1098765 .0077483 14.18 0.000 .0946901 .125063

difference | -.0223849 .0093048 -2.41 0.016 -.040622 -.0041477

explained | -.0071848 .0083112 -0.86 0.387 -.0234745 .0091048

unexplained | -.0152 .0130407 -1.17 0.244 -.0407593 .0103592

-------------+----------------------------------------------------------------

explained |

mage2 | .0007257 .0008659 0.84 0.402 -.0009714 .0024228

mage3 | .0000595 .0002774 0.21 0.830 -.0004842 .0006032

medu1 | -.0031035 .0036474 -0.85 0.395 -.0102523 .0040453

medu2 | -.0003042 .0013651 -0.22 0.824 -.0029797 .0023713

rural1 | .0033164 .0064383 0.52 0.606 -.0093025 .0159353

notwork | .000221 .0003524 0.63 0.531 -.0004696 .0009116

media | -.0073856 .0045311 -1.63 0.103 -.0162663 .0014952

water | -.000291 .002377 -0.12 0.903 -.0049497 .0043678

toilety | .0048836 .0051168 0.95 0.340 -.0051452 .0149123

mar1 | -.0001018 .0004694 -0.22 0.828 -.0010218 .0008182

mar2 | -.0000287 .000119 -0.24 0.809 -.0002619 .0002045

sex | .0005344 .0004082 1.31 0.190 -.0002656 .0013345

age | -6.09e-06 .0003204 -0.02 0.985 -.000634 .0006218

weightm2 | -.00004 .0001042 -0.38 0.701 -.0002444 .0001643

weightm3 | -.0028904 .0010947 -2.64 0.008 -.005036 -.0007448

border1 | -.0001557 .0004969 -0.31 0.754 -.0011295 .0008182

border2 | .0002093 .0002622 0.80 0.425 -.0003046 .0007233

border3 | .0000179 .0000906 0.20 0.843 -.0001597 .0001956

bint2 | .0001972 .0008557 0.23 0.818 -.0014798 .0018743

bint3 | 0 (omitted)

com_zsesc52 | .0030077 .0024053 1.25 0.211 -.0017066 .0077221

com_zsesc53 | -1.78e-06 .0001988 -0.01 0.993 -.0003914 .0003879

com_zsesc54 | -.0023366 .0039253 -0.60 0.552 -.01003 .0053569

com_zsesc55 | -.0037124 .0073316 -0.51 0.613 -.0180821 .0106573

-------------+----------------------------------------------------------------

unexplained |

mage2 | .008098 .0185 0.44 0.662 -.0281613 .0443573

mage3 | .0036129 .0107559 0.34 0.737 -.0174683 .0246942

medu1 | .0002971 .0087708 0.03 0.973 -.0168934 .0174877

medu2 | -.0037842 .0064887 -0.58 0.560 -.0165017 .0089334

rural1 | -.0305627 .0442808 -0.69 0.490 -.1173515 .056226

notwork | -.0056545 .0145418 -0.39 0.697 -.0341559 .0228468

media | -.0124769 .0095577 -1.31 0.192 -.0312097 .006256

water | .0382045 .0256323 1.49 0.136 -.0120339 .0884429

toilety | -.0287545 .0167384 -1.72 0.086 -.0615612 .0040523

mar1 | -.0002241 .0001834 -1.22 0.222 -.0005835 .0001352

mar2 | -.0735506 .0845037 -0.87 0.384 -.2391749 .0920736

sex | -.0214514 .0144233 -1.49 0.137 -.0497206 .0068177

age | -.0251296 .0222134 -1.13 0.258 -.0686669 .0184078

weightm2 | -.0017178 .0018488 -0.93 0.353 -.0053414 .0019058

weightm3 | -.0023296 .0028564 -0.82 0.415 -.0079281 .0032688

border1 | .002103 .0060509 0.35 0.728 -.0097565 .0139626

border2 | .0022809 .0067243 0.34 0.734 -.0108985 .0154603

border3 | .0052253 .0056892 0.92 0.358 -.0059252 .0163759

bint2 | -.0074592 .0162669 -0.46 0.647 -.0393417 .0244233

bint3 | -.0099727 .0100308 -0.99 0.320 -.0296327 .0096873

com_zsesc52 | .0178295 .0110579 1.61 0.107 -.0038435 .0395025

com_zsesc53 | .0288984 .0175546 1.65 0.100 -.0055079 .0633048

com_zsesc54 | .0442729 .0248188 1.78 0.074 -.0043711 .0929169

com_zsesc55 | .0513201 .0282264 1.82 0.069 -.0040026 .1066428

_cons | .005725 .1115133 0.05 0.959 -.2128371 .2242872

------------------------------------------------------------------------------

19

(water mar1 border1 dropped from model 1)

(mar1 bint3 dropped from model 2)

(model 2 has zero variance coefficients)

(mar1 border1 dropped from pooled model)

(water mar1 border1 missing in model 1; assumed zero)

(mar1 bint3 missing in model 2; assumed zero)

(mar1 border1 missing in pooled model; assumed zero)

Blinder-Oaxaca decomposition Number of obs = 3602

Model = logit

Group 1: poor = 0 N of obs 1 = 1614

Group 2: poor = 1 N of obs 2 = 1988

------------------------------------------------------------------------------

| Robust

sam | Coef. Std. Err. z P>|z| [95% Conf. Interval]

-------------+----------------------------------------------------------------

overall |

group_1 | .0099133 .0023493 4.22 0.000 .0053087 .0145178

group_2 | .0201207 .0034064 5.91 0.000 .0134443 .0267971

difference | -.0102075 .004138 -2.47 0.014 -.0183177 -.0020972

explained | -.0052494 .0036878 -1.42 0.155 -.0124773 .0019785

unexplained | -.004958 .0056194 -0.88 0.378 -.0159718 .0060557

-------------+----------------------------------------------------------------

explained |

mage2 | .0002956 .0002954 1.00 0.317 -.0002832 .0008745

mage3 | .0002414 .0002434 0.99 0.321 -.0002357 .0007186

medu1 | .0006804 .0011324 0.60 0.548 -.001539 .0028998

medu2 | .0001902 .0009836 0.19 0.847 -.0017375 .002118

rural1 | .0008124 .0023001 0.35 0.724 -.0036957 .0053205

notwork | -.0002172 .0003231 -0.67 0.501 -.0008504 .000416

media | -.0000629 .0011531 -0.05 0.957 -.002323 .0021973

water | -.0002591 .0013245 -0.20 0.845 -.0028551 .0023369

toilety | -.000952 .0022384 -0.43 0.671 -.0053392 .0034351

mar1 | 0 (omitted)

mar2 | -.0000131 .0001111 -0.12 0.906 -.0002307 .0002046

sex | -.0001001 .0001232 -0.81 0.416 -.0003415 .0001413

age | .0000152 .0003312 0.05 0.963 -.000634 .0006644

weightm2 | -.0000715 .0001012 -0.71 0.480 -.0002699 .0001268

weightm3 | -.0000959 .0001036 -0.93 0.355 -.0002989 .0001071

border1 | 0 (omitted)

border2 | .0003602 .0003378 1.07 0.286 -.0003019 .0010222

border3 | -.0000186 .0001386 -0.13 0.893 -.0002903 .000253

bint2 | .0001496 .000677 0.22 0.825 -.0011772 .0014765

bint3 | -.0002418 .0006386 -0.38 0.705 -.0014935 .0010099

com_zsesc52 | .000569 .001314 0.43 0.665 -.0020064 .0031443

com_zsesc53 | -.0000211 .0001737 -0.12 0.903 -.0003615 .0003193

com_zsesc54 | -.001914 .0017765 -1.08 0.281 -.005396 .001568

com_zsesc55 | -.0045961 .003176 -1.45 0.148 -.0108209 .0016287

-------------+----------------------------------------------------------------

unexplained |

mage2 | -.0099375 .0227151 -0.44 0.662 -.0544582 .0345832

mage3 | -.0123905 .0275476 -0.45 0.653 -.0663827 .0416018

medu1 | .0044807 .0095771 0.47 0.640 -.0142901 .0232515

medu2 | .0066005 .0188867 0.35 0.727 -.0304168 .0436178

rural1 | -.0216141 .0579052 -0.37 0.709 -.1351062 .091878

notwork | -.0015624 .0046088 -0.34 0.735 -.0105955 .0074707

media | .0002631 .0098304 0.03 0.979 -.0190042 .0195304

water | .0011341 .0090505 0.13 0.900 -.0166046 .0188727

toilety | .0039633 .0098445 0.40 0.687 -.0153317 .0232582

mar1 | 0 (omitted)

mar2 | -.0371886 .0757028 -0.49 0.623 -.1855634 .1111862

sex | .0061884 .0128463 0.48 0.630 -.01899 .0313667

age | -.0130034 .0209876 -0.62 0.536 -.0541383 .0281315

weightm2 | -.0001041 .0023976 -0.04 0.965 -.0048032 .0045951

weightm3 | .0010194 .0020326 0.50 0.616 -.0029645 .0050033

border1 | -.0023554 .0055108 -0.43 0.669 -.0131564 .0084456

border2 | .0010242 .0043941 0.23 0.816 -.0075881 .0096366

border3 | -.0016791 .0044442 -0.38 0.706 -.0103897 .0070314

bint2 | .0018757 .0104838 0.18 0.858 -.0186722 .0224236

bint3 | .0054341 .0129467 0.42 0.675 -.0199409 .0308092

com_zsesc52 | -.036196 .0762959 -0.47 0.635 -.1857332 .1133411

com_zsesc53 | -.0577871 .1221409 -0.47 0.636 -.2971789 .1816047

com_zsesc54 | -.1071531 .227553 -0.47 0.638 -.5531487 .3388425

com_zsesc55 | -.1168498 .2495567 -0.47 0.640 -.605972 .3722724

_cons | .3808796 .817136 0.47 0.641 -1.220677 1.982437

------------------------------------------------------------------------------

20

(medu1 water mar1 border1 dropped from model 1)

(medu1 rural1 border1 com_zsesc52 com_zsesc54 dropped from model 2)

(medu1 bint2 dropped from pooled model)

(medu1 water mar1 border1 missing in model 1; assumed zero)

(medu1 rural1 border1 com_zsesc52 com_zsesc54 missing in model 2; assumed zero)

(medu1 bint2 missing in pooled model; assumed zero)

Blinder-Oaxaca decomposition Number of obs = 4258

Model = logit

Group 1: poor = 0 N of obs 1 = 2445

Group 2: poor = 1 N of obs 2 = 1813

------------------------------------------------------------------------------

| Robust

sam | Coef. Std. Err. z P>|z| [95% Conf. Interval]

-------------+----------------------------------------------------------------

overall |

group_1 | .0102249 .0020337 5.03 0.000 .0062391 .0142108

group_2 | .0170987 .0030419 5.62 0.000 .0111366 .0230608

difference | -.0068738 .0036591 -1.88 0.060 -.0140455 .000298

explained | -.0051272 .0065444 -0.78 0.433 -.0179539 .0076995

unexplained | -.0017466 .0079638 -0.22 0.826 -.0173554 .0138621

-------------+----------------------------------------------------------------

explained |

mage2 | .000278 .0002425 1.15 0.252 -.0001973 .0007533

mage3 | .0000142 .0000593 0.24 0.810 -.000102 .0001304

medu1 | 0 (omitted)

medu2 | .0006468 .00142 0.46 0.649 -.0021364 .00343

rural1 | .0123737 .005343 2.32 0.021 .0019016 .0228458

notwork | -.0004684 .0006796 -0.69 0.491 -.0018003 .0008635

media | -.0042922 .0023528 -1.82 0.068 -.0089035 .0003192

water | .0003492 .0018811 0.19 0.853 -.0033376 .004036

toilety | -.0018283 .0034366 -0.53 0.595 -.008564 .0049074

mar1 | -.0003816 .0005711 -0.67 0.504 -.001501 .0007378

mar2 | .000053 .0002477 0.21 0.831 -.0004326 .0005385

sex | .0000313 .0001157 0.27 0.787 -.0001954 .000258

age | -.0000667 .0000814 -0.82 0.413 -.0002263 .0000929

weightm2 | -.0000157 .0000459 -0.34 0.733 -.0001057 .0000744

weightm3 | -.0001162 .0001079 -1.08 0.281 -.0003277 .0000952

border1 | -.0004758 .0003776 -1.26 0.208 -.0012158 .0002643

border2 | -.0001746 .0003374 -0.52 0.605 -.0008359 .0004868

border3 | .0000575 .0001216 0.47 0.636 -.0001809 .0002959

bint2 | 0 (omitted)

bint3 | -.0000318 .0000697 -0.46 0.648 -.0001685 .0001048

com_zsesc52 | -.002242 .0018818 -1.19 0.233 -.0059302 .0014462

com_zsesc53 | -.0004984 .0003576 -1.39 0.163 -.0011993 .0002025

com_zsesc54 | -.0034292 .0022743 -1.51 0.132 -.0078867 .0010283

com_zsesc55 | -.0049101 .0043012 -1.14 0.254 -.0133403 .00352

-------------+----------------------------------------------------------------

unexplained |

mage2 | -.000433 .0018917 -0.23 0.819 -.0041407 .0032746

mage3 | -.0000956 .0007712 -0.12 0.901 -.0016071 .001416

medu1 | 0 (omitted)

medu2 | -.0005969 .0021722 -0.27 0.783 -.0048543 .0036605

rural1 | -.0056179 .0208305 -0.27 0.787 -.0464449 .0352092

notwork | .0001068 .0009587 0.11 0.911 -.0017722 .0019857

media | -.0001745 .0012868 -0.14 0.892 -.0026967 .0023476

water | -.0001812 .0015594 -0.12 0.908 -.0032375 .0028751

toilety | -.001692 .0062531 -0.27 0.787 -.0139478 .0105639

mar1 | .0000252 .0001111 0.23 0.820 -.0001925 .000243

mar2 | .0003351 .0033683 0.10 0.921 -.0062667 .0069369

sex | .0010532 .0039272 0.27 0.789 -.006644 .0087504

age | -.0030621 .0112437 -0.27 0.785 -.0250994 .0189752

weightm2 | -.0000404 .0003075 -0.13 0.895 -.0006432 .0005623

weightm3 | .0001416 .0005256 0.27 0.788 -.0008886 .0011719

border1 | .0001304 .0004632 0.28 0.778 -.0007775 .0010384

border2 | -.0006355 .0023374 -0.27 0.786 -.0052168 .0039458

border3 | -.000869 .0031235 -0.28 0.781 -.0069908 .0052529

bint2 | .0019619 .0070841 0.28 0.782 -.0119226 .0158465

bint3 | .0032105 .0116392 0.28 0.783 -.0196019 .0260228

com_zsesc52 | .0000105 .0001285 0.08 0.935 -.0002412 .0002623

com_zsesc53 | .0011201 .0040723 0.28 0.783 -.0068615 .0091016

com_zsesc54 | .0013092 .0047607 0.28 0.783 -.0080216 .0106401

com_zsesc55 | .0022988 .0082957 0.28 0.782 -.0139605 .0185581

_cons | -.0000519 .0058249 -0.01 0.993 -.0114685 .0113646

------------------------------------------------------------------------------

.

.

. di "`country_first'"

Zimbabwe

. di "`country_reshape'"

Angola Bangladesh Burundi CDR Cameroun Ethiopia Ghana India Kenya Lesotho Mali Mozambique Namibia Niger Nigeria Pakistan

> Senegal Timor Uganda Zimbabwe

. di "`country_keep'"

Angola* Bangladesh* Burundi* CDR* Cameroun* Ethiopia* Ghana* India* Kenya* Lesotho* Mali* Mozambique* Namibia* Niger* Nigeri

> a* Pakistan* Senegal* Timor* Uganda* Zimbabwe*

.

. keep `country_keep'

. gen id = _n

. reshape long `country_reshape', i(id) j(cat)

(note: j = 1 2 3 4 5 6 7 8 9 10 11 12 13 14 15 16 17 18 19 20 21 22 23 24 25 26 27 28 29 30 31 32 33 34 35 36 37 38 39 40 41 42 43 44 45 46 47 48 49 50

> 51 52 53 54)

Data wide -> long

-----------------------------------------------------------------------------

Number of obs. 363561 -> 2.0e+07

Number of variables 1081 -> 22

j variable (54 values) -> cat

xij variables:

Angola1 Angola2 ... Angola54 -> Angola

Bangladesh1 Bangladesh2 ... Bangladesh54 -> Bangladesh

Burundi1 Burundi2 ... Burundi54 -> Burundi

CDR1 CDR2 ... CDR54 -> CDR

Cameroun1 Cameroun2 ... Cameroun54 -> Cameroun

Ethiopia1 Ethiopia2 ... Ethiopia54 -> Ethiopia

Ghana1 Ghana2 ... Ghana54 -> Ghana

India1 India2 ... India54 -> India

Kenya1 Kenya2 ... Kenya54 -> Kenya

Lesotho1 Lesotho2 ... Lesotho54 -> Lesotho

Mali1 Mali2 ... Mali54 -> Mali

Mozambique1 Mozambique2 ... Mozambique54 -> Mozambique

Namibia1 Namibia2 ... Namibia54 -> Namibia

Niger1 Niger2 ... Niger54 -> Niger

Nigeria1 Nigeria2 ... Nigeria54 -> Nigeria

Pakistan1 Pakistan2 ... Pakistan54 -> Pakistan

Senegal1 Senegal2 ... Senegal54 -> Senegal

Timor1 Timor2 ... Timor54 -> Timor

Uganda1 Uganda2 ... Uganda54 -> Uganda

Zimbabwe1 Zimbabwe2 ... Zimbabwe54 -> Zimbabwe

-----------------------------------------------------------------------------

. keep if !missing(`country_first')

(19632240 observations deleted)

.

. gen str factorLabel = ""

(54 missing values generated)

. local j "1"

. foreach name of local names {

2. di "`name'"

3. di "`j'"

4. replace factorLabel = "`name'" in `j'

5. local ++j

6. }

overall:group_1

1

factorLabel was str1 now str15

(1 real change made)

overall:group_2

2

(1 real change made)

overall:difference

3

factorLabel was str15 now str18

(1 real change made)

overall:explained

4

(1 real change made)

overall:unexplained

5

factorLabel was str18 now str19

(1 real change made)

explained:mage2

6

(1 real change made)

explained:mage3

7

(1 real change made)

explained:medu1

8

(1 real change made)

explained:medu2

9

(1 real change made)

explained:rural1

10

(1 real change made)

explained:notwork

11

(1 real change made)

explained:media

12

(1 real change made)

explained:water

13

(1 real change made)

explained:toilety

14

(1 real change made)

explained:mar1

15

(1 real change made)

explained:mar2

16

(1 real change made)

explained:sex

17

(1 real change made)

explained:age

18

(1 real change made)

explained:weightm2

19

(1 real change made)

explained:weightm3

20

(1 real change made)

explained:border1

21

(1 real change made)

explained:border2

22

(1 real change made)

explained:border3

23

(1 real change made)

explained:bint2

24

(1 real change made)

explained:bint3

25

(1 real change made)

explained:com_zsesc52

26

factorLabel was str19 now str21

(1 real change made)

explained:com_zsesc53

27

(1 real change made)

explained:com_zsesc54

28

(1 real change made)

explained:com_zsesc55

29

(1 real change made)

unexplained:mage2

30

(1 real change made)

unexplained:mage3

31

(1 real change made)

unexplained:medu1

32

(1 real change made)

unexplained:medu2

33

(1 real change made)

unexplained:rural1

34

(1 real change made)

unexplained:notwork

35

(1 real change made)

unexplained:media

36

(1 real change made)

unexplained:water

37

(1 real change made)

unexplained:toilety

38

(1 real change made)

unexplained:mar1

39

(1 real change made)

unexplained:mar2

40

(1 real change made)

unexplained:sex

41

(1 real change made)

unexplained:age

42

(1 real change made)

unexplained:weightm2

43

(1 real change made)

unexplained:weightm3

44

(1 real change made)

unexplained:border1

45

(1 real change made)

unexplained:border2

46

(1 real change made)

unexplained:border3

47

(1 real change made)

unexplained:bint2

48

(1 real change made)

unexplained:bint3

49

(1 real change made)

unexplained:com_zsesc52

50

factorLabel was str21 now str23

(1 real change made)

unexplained:com_zsesc53

51

(1 real change made)

unexplained:com_zsesc54

52

(1 real change made)

unexplained:com_zsesc55

53

(1 real change made)

unexplained:_cons

54

(1 real change made)

.

. drop id cat

. order factorLabel

.

. foreach var of varlist `country_reshape' {

2. gen per_`var' = (`var' / `var'[3])*100

3. }

.

. outsheet using decomp_poorjune.csv, replace comma
