# Supplementary material for: Mind the gap: What explains the poor-non-poor inequalities in severe wasting among under-five children in low- and middle-income countries? Compositional and structural characteristics
Source: PLoS One. 2020 Nov 3;15(11):e0241416. doi: 10.1371/journal.pone.0241416 (PMC7608875; doi:10.1371/journal.pone.0241416)
Supplement: Attachment — Submitted filename: Response Plos One SAM Poverty.docx [file pone.0241416.s002.docx]

The Editor,

Plos One

Attention:

Bruno Masquelier, PhD
Academic Editor
PLOS ONE

RE: PONE-D-19-28363
Mind the Gap: What explains the poor-non-poor inequalities in severe acute malnutrition among under-five children in low and middle-Income countries? Compositional and structural characteristics

We the authors of above-mentioned paper appreciate the efforts and comments of the academic editor. We have addressed all these comments. A point-by-point response to the issues in our revised manuscript is listed below. We wish to resubmit for your kind reconsideration

This article focuses on a very important topic, but it has important flaws that should be addressed before being submitted to external reviewers. In particular, the article does not meet the third criterion for publication in PLOS ONE, requiring that “experiments, statistics, and other analyses are performed to a high technical standard and are described in sufficient detail”.

The main problem is that the description of the methods used is too brief, and the reader has difficulty interpreting the results.

We have reviewed the entire methodology

For example, Figure 1 mentions random effects while the description of the methods does not include any reference to mixed-effects or hierarchical models.

We have provided additional information on all Figures and Tables to enable easy readership

How were the estimates presented in Figure 1 obtained and why don’t they match the descriptive statistics?

We have explained the computations. We regret the differences between the estimates in Figure 1 and the descriptive statistics. It was due to weighting. The estimates in the Figures and the Tables are now the same

There is also a problem of consistency in the presentation, since children are first divided into two groups (poor, non-poor) when presenting descriptive statistics, and then variables at different levels (individual, contextual) are used to explain the gap between poor and non-poor, but some of the variables (such as the type of sanitation or source of drinking water) are used for constructing the wealth index, while others are not involved in the construction of this index.

We agreed with these comments. We included whether the sources of drinking water and toilet types are improved or not in the descriptive studies in Tables 1 and 2 to show the particular roles (differentials) that these variables could play. We dropped them in the decomposition analysis in our reviewed analysis.

Authors should also carefully decide which results to present and facilitate their interpretation in the main text. For example, Figure 4 and Figure 5 present the same information twice in a different way.

We appreciate this point. We have adopted the adjusted Figure 5

But how can we interpret contributions of -870% in Lesotho, for example?

This value is an exception to all other values. It shows the magnitude of the unexplained variations between the children from poor households and those from non-poor households differed greatly in the development of SAM to the tune of 870%. That is, the contributions of neighbourhood socio-economic status varied widely between the two groups of children.

Finally, DHS allow measuring the prevalence of wasting based on weight-for-height Z-scores, stunting based on height-for-age Z-scores, and the proportion of underweight children based on weight-for-age Z-scores. Yet, the authors refer to SAM, which also includes "visible severe wasting or the presence of nutritional oedema" in their analysis. Should the paper be re-framed around wasting instead of SAM? Overall the estimates presented in this paper are very close to the % of children that are severely wasted in DHS.

Our study is focussed on SAM and we adopted the definitions of WHO in our computations. We believe that SAM should be retained as it connotes advanced wasting <https://www.who.int/nutrition/topics/severe_malnutrition/en/>

In addition to these changes to the methods and results, the authors are invited to consult with a copy editor when revising their paper to smooth over some rough spots in the exposition (e.g. “a difference of 30 children among 1000 who have SAM are from poor mothers”).

Thank you, we have engaged the services of language editor and corrected this and similar errors.
