# Supplementary material for: Mind the gap: What explains the poor-non-poor inequalities in severe wasting among under-five children in low- and middle-income countries? Compositional and structural characteristics
Source: PLoS One. 2020 Nov 3;15(11):e0241416. doi: 10.1371/journal.pone.0241416 (PMC7608875; doi:10.1371/journal.pone.0241416)
Supplement: Attachment — Submitted filename: Prose 0ne OB decompe final.docx [file pone.0241416.s003.docx]

Dear Editor and authors

I have gone point by point regarding the things I asked them to do. I am glad to say that they have addressed all the things. A job well done

All the best

Reviewer
